# Electron‐Mediator‐Free Microfluidic Photocatalytic Coenzyme Regeneration with 100% Conversion Efficiency within 126 S

**DOI:** 10.1002/advs.202513720

**Published:** 2025-11-07

**Authors:** Yao Chai, Liang Wan, Zirui Pang, Xu Li, Zixuan Jia, Heng Jiang, Chi Chung Tsoi, Huaping Jia, Jinni Shen, Zizhong Zhang, Jinlin Long, Fengjia Xie, Yanmei Chen, Xuming Zhang

**Affiliations:** ^1^ Department of Applied Physics The Hong Kong Polytechnic University Hong Kong Kowloon 999077 China; ^2^ Photonics Research Institute (PRI) The Hong Kong Polytechnic University Hong Kong Kowloon 999077 China; ^3^ State Key Laboratory of Photocatalysis on Energy and Environment College of Chemistry Fuzhou University Fuzhou 350116 China

**Keywords:** BiOBr nanosheets, electron mediators, microfluidics, NAD(P)H regeneration, photocatalysis

## Abstract

Microfluidic systems enhance photocatalytic efficiency through superior mass/energy transfer and precise parameter control. Here, an electron‐mediator‐free microfluidic platform is designed for photocatalytic coenzyme NAD(P)H regeneration by integrating ultrathin BiOBr nanosheets. This architecture enables direct electron–proton coupling, achieving 100% NAD^+^ conversion within 126 s with exceptional selectivity (72.30%) for bioactive 1,4‐NADH. Continuous operation over 32 h shows no activity decay, demonstrating unparalleled stability. This study establishes a benchmark for designing integrated photocatalytic systems and highlights their broad potential for applications in biocatalysis, synthetic biology, and renewable energy.

## Graphical abstract

Discover a groundbreaking mediator‐free photocatalytic method for coenzyme NAD(P)H regeneration using BiOBr nanosheets in a microfluidic chip. This study achieves 100% NAD^+^ to NADH conversion in 126 s, maintaining >87% efficiency over 32 h. Explore how photocatalysis and microfluidics combine to offer a sustainable, scalable solution for green chemistry.

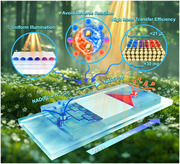

## 1 Introduction

Efficient coenzyme regeneration is a core technology in biocatalysis and sustainable chemistry, with significant applications in enzymatic reactions and synthetic biology.^[^ ^1^, ^2^ ^]^ Nicotinamide adenine dinucleotide (NADH) and its phosphorylated form (NADPH) play a critical role in redox reactions essential for cellular metabolism.^[^ ^3^, ^4^ ^]^ However, traditional regeneration methods rely on stoichiometric chemical reagents or complex reaction systems, which consume significant resources and often generate undesirable byproducts.^[^ ^5^ ^]^ Photocatalysis emerges as a promising alternative, utilizing light energy to drive coenzyme regeneration while reducing environmental impact.^[^ ^6^, ^7^ ^]^ However, existing photocatalytic systems face challenges such as low electron transfer efficiency, limited conversion.^[^ ^8^, ^9^ ^]^ Additionally, their reliance on electron mediators increases catalytic costs and complicates system integration, significantly restricting their practical applications in efficient energy conversion and chemical synthesis.^[^ ^10^, ^11^ ^]^

In recent years, microfluidic technology has emerged as an innovative approach for optimizing photocatalytic processes.^[^ ^12^ ^]^ Microfluidic systems have gained prominence due to their unique advantages, including efficient mass and energy transfer, precise control over reaction conditions, and greatly reduced reagent consumption, rendering them highly promising for catalytic applications.^[^ ^13^, ^14^ ^]^ For instance, microfluidic technology has demonstrated potential through enhanced reaction kinetics and selectivity in processes such as photocatalytic hydrogen production, CO_2_ reduction, and CH_4_ oxidation.^[^ ^15^, ^16^, ^17^ ^]^ These successful applications provide valuable insights for coenzyme regeneration research. In the context of coenzyme regeneration, microfluidic systems enable an optimal residence time of reactants on the catalyst surface via precise fluid control, which not only enhances regeneration efficiency but also improves product selectivity.^[^ ^18^ ^]^ By integrating advanced catalytic materials such as g‐C_3_N_4_ nanosheets into the microfluidic platform, it is possible to not only optimize the separation efficiency of photogenerated electron–hole pairs but also strengthen the specificity of interactions with coenzyme molecules through surface modification. Furthermore, the modular design of such systems allows the integration of light sources, temperature control units, and online monitoring components into a single microfluidic platform, effectively constructing an “on‐chip photocatalytic factory” that significantly lowers operating costs and energy consumption.^[^ ^19^, ^20^ ^]^ This integrated strategy injects new impetus into the development of efficient and economical coenzyme regeneration systems, substantially boosting their potential for practical application.

In this study, we presented a novel electron‐mediator‐free microfluidic system for the photocatalytic regeneration of coenzyme NAD(P)H. By integrating ultrathin BiOBr nanosheets into a microfluidic chip, the system enabled direct electron–proton coupling without requiring electron mediators. This innovative design achieved 100% NAD^+^ conversion in just 126 s while maintaining high selectivity (72.30%) for the biologically active 1,4‐NADH isomer. Furthermore, the system exhibited outstanding operational stability, sustaining consistent catalytic performance over 36 h of continuous operation without significant loss of activity or byproduct formation. These results highlighted the robustness and efficiency of this system, establishing it as a benchmark for integrated photocatalytic system development. This work not only advanced the field of coenzyme regeneration but also underscored the transformative potential of microfluidics in biocatalysis, synthetic biology, and renewable energy. By addressing key challenges associated with conventional systems, this study paved the way for designing more efficient, sustainable, and scalable catalytic technologies.

## 2 Results and Discussion

**Figure 1:**
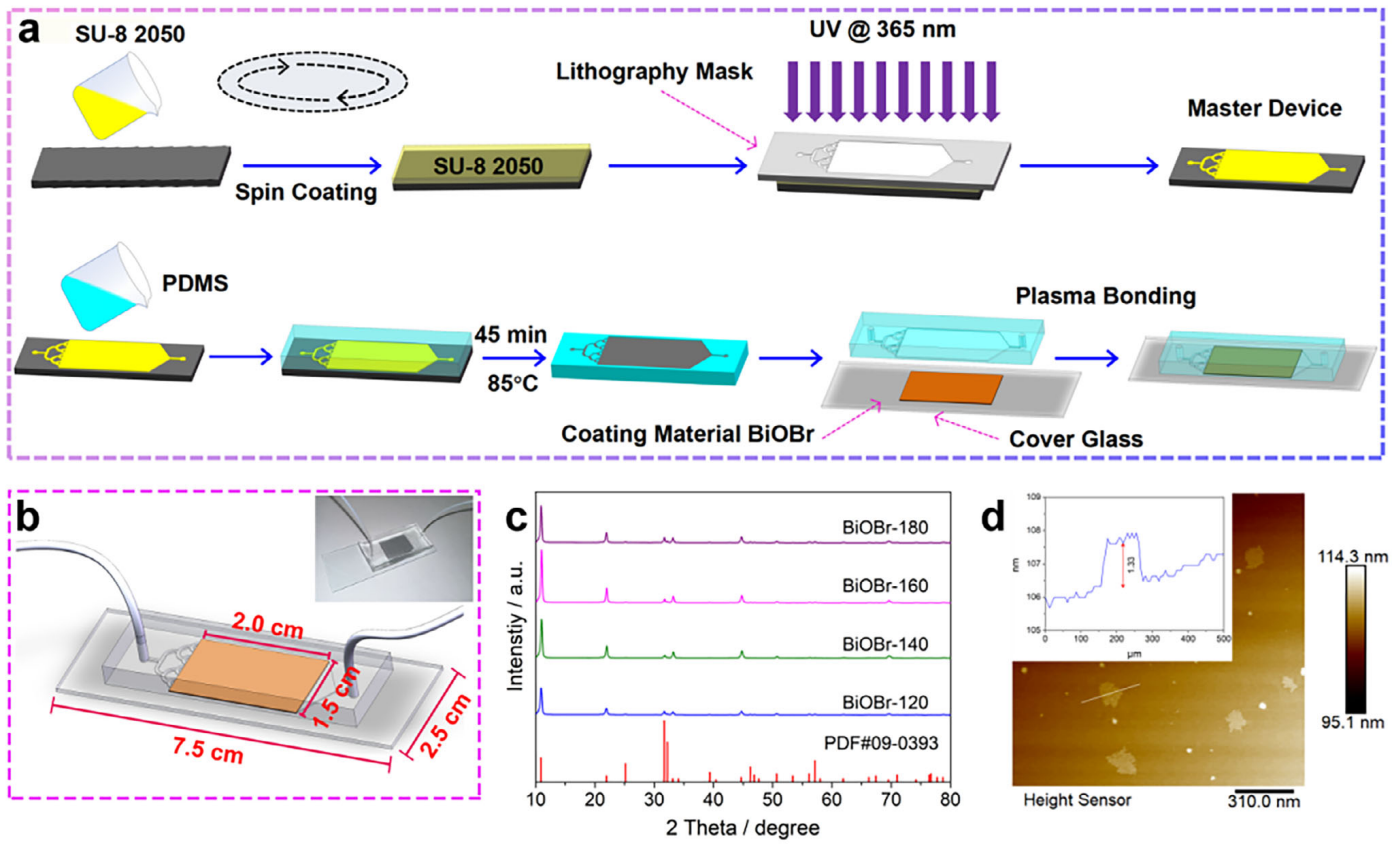
a) Schematic illustration of the preparation process for the microfluidic chip designed for photocatalytic regeneration of the coenzyme NAD(P)H, highlighting the photolithography and soft lithography steps. b) Schematic representation of the microfluidic chip used for photocatalytic NAD(P)H regeneration, with an inset showing an actual photograph of the chip. c) XRD patterns of BiOBr synthesized at varying temperatures. d) AFM image of BiOBr‐180 nanosheets.

The microfluidic chip for coenzyme regeneration was fabricated using photolithography and soft lithography processes, as illustrated in **Figure** **1**a. During photolithography, SU‐8 2050 photoresist and a 4‐inch silicon wafer were used as the substrate. The photolithography process included coating, prebaking, exposure, postbaking, development, and curing steps. First, the channel structure pattern from the mask was transferred onto the SU‐8 photoresist coated on the silicon wafer through photolithography. After development, the mold structure corresponding to the transparent regions of the mask was obtained. Next, polydimethylsiloxane (PDMS) and a curing agent were mixed at a 10:1 mass ratio, and the mixed PDMS material was poured onto the silicon wafer mold. The mold and PDMS were heated in an oven at 85 °C for 1 h to cure. The cured PDMS was then removed with tweezers and cut according to the mold structure to form the PDMS microchannel structure. Finally, oxygen plasma (O_2_ plasma) treatment was used to clean the surfaces of the PDMS channels and the glass slide, and the two were bonded to complete the assembly of the microfluidic chip. It is worth noting that the glass slide was uniformly coated with a layer of photocatalyst to support subsequent photocatalytic reactions. Figure 1b shows the structure of the complete microfluidic chip, which measures 7.5 cm × 2.5 cm. The photocatalytic reaction area within the chip had dimensions of 2.0 cm × 1.5 cm × 0.007 cm. A coating of 30 mg of photocatalyst was applied uniformly to the reaction area, yielding a loading of ≈10 mg cm^−2^.

The crystal structure of the synthesized BiOBr photocatalyst was analyzed using X‐ray powder diffraction (XRD). The results show that all diffraction peaks match the standard peaks of tetragonal BiOBr (Figure 1c).^[^ ^21^ ^]^ Notably, as the preparation temperature of the catalyst increases, the intensity of the diffraction peak corresponding to the (0 0 1) crystal plane significantly enhances, indicating an improved preferential orientation of the (0 0 1) plane in BiOBr nanosheets. This phenomenon suggests that the material primarily grows along the (0 0 1) direction, leading to an increased exposure ratio of the (0 0 1) plane. Scanning electron microscopy characterization confirmed that this series of photocatalysts exhibited a typical 2D nanosheet morphology (Figure S1, Supporting Information). Transmission electron microscopy further verified the presence of this structure (Figure S2, Supporting Information). Atomic force microscopy analysis revealed that the nanosheet thickness progressively decreased with increasing catalyst synthesis temperature (Figure S3, Supporting Information). This trend resulted from the synthesis process, which was typically accompanied by crystal growth and material rearrangement under high‐temperature conditions. At elevated temperatures, enhanced thermal motion reduced the lattice energy of the nanosheets, prompting the system to optimize its structure by minimizing energy, thereby leading to a decrease in thickness. The results indicated that BiOBr‐180 exhibited the thinnest nanosheets, with a thickness of ≈1.33 nm. Although this value was slightly larger than the theoretical thickness of a single‐layer BiOBr nanosheet along the [0 0 1] direction (0.811 nm, half the unit cell size along the *c*‐axis), it remained relatively close (Figure 1d; Figure S4, Supporting Information). The discrepancy may have arisen from interlayer interactions, surface adsorption, or lattice distortion.^[^ ^22^ ^]^ The ultrathin 2D structure significantly enhanced the specific surface area, exposed more active sites, improved electron–hole separation efficiency, and boosted photocatalytic performance, ultimately facilitating the photocatalytic reaction.

**Figure 2:**
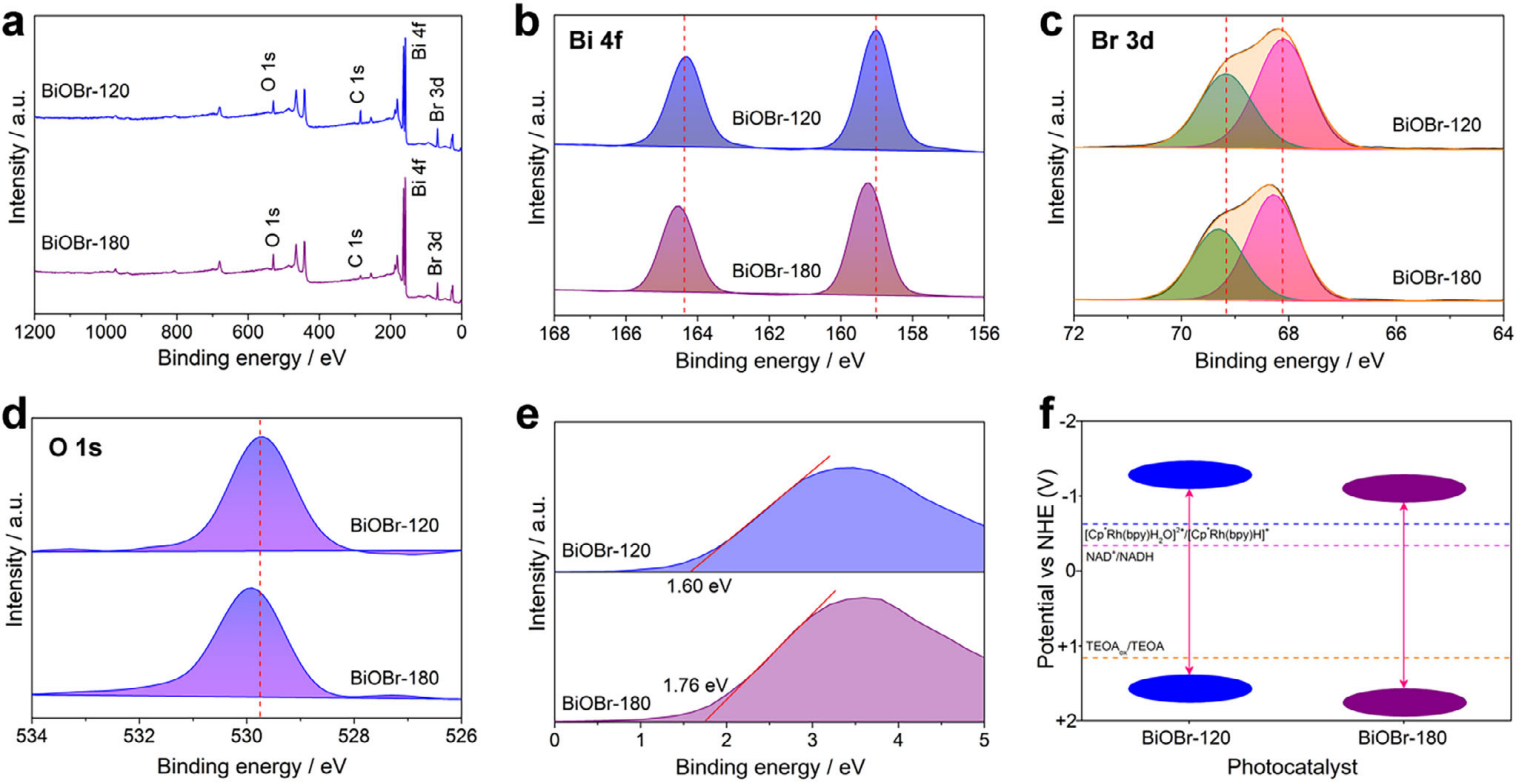
XPS survey spectra of BiOBr: a) Full spectrum, b) Bi 4f, c) Br 3d, and d) O 1s. e) Valence band XPS spectra of BiOBr‐120 and BiOBr‐180 photocatalysts. f) Band positions of BiOBr‐120 and BiOBr‐180.

X‐ray photoelectron spectroscopy (XPS) analysis of BiOBr nanosheets confirms their chemical composition and elucidates the valence states of constituent elements, with all spectra referenced to adventitious carbon (C 1s, 284.8 eV). The survey spectra verify the presence of Bi, O, and Br in both BiOBr‐120 and BiOBr‐180 (**Figure** **2**a). High‐resolution Bi 4f spectra reveals spin‐orbit doublets (Bi 4f_7/2_ and Bi 4f_5/2_) at 159.02 and 164.33 eV for BiOBr‐120, characteristic of Bi^3+^ (Figure 2b).^[^ ^23^ ^]^ A discernible positive shift in these peaks for BiOBr‐180 indicates an altered chemical environment around Bi^3+^. Similarly, the Br 3d spectra exhibits peaks (Br 3d_5/2_ and Br 3d_3/2_) at 68.11 and 69.17 eV for BiOBr‐120, shifting to 68.28 and 69.30 eV for BiOBr‐180, both align with Br^−^ in BiOBr (Figure 2c).^[^ ^24^ ^]^ The O 1s core level shows a binding energy of 529.74 eV for BiOBr‐120 and 529.94 eV for BiOBr‐180, consistent with lattice oxygen despite the shift (Figure 2d).^[^ ^25^ ^]^ Valence‐band XPS spectra, analyzed by linear extrapolation, yield valence band maxima (VBM) positions of +1.60 eV and +1.76 eV (relative to the Fermi level) for BiOBr‐120 and BiOBr‐180, respectively (Figure 2e).^[^ ^26^ ^]^ Combined with UV–vis diffuse reflectance spectra (Figure S5, Supporting Information), which give bandgaps of 2.75–2.80 eV, the conduction band minima (CBM) are calculated to be −1.15 and −1.02 eV (E_g_ = E_VBM_ – E_CBM_). Critically, the CBM energies of both materials are positioned more negatively than the reduction potentials of the relevant electron mediator and NAD^+^/NAD(P)H. Concurrently, their VBM energies lie more positively than the oxidation potential of triethanolamine (TEOA). This energetic alignment confirms that photoexcited electrons possess sufficient reducing power for NAD^+^ reduction, while photogenerated holes can efficiently oxidize TEOA, fulfilling the thermodynamic requirements for sustained photocatalytic cycling (Figure 2f).

**Figure 3:**
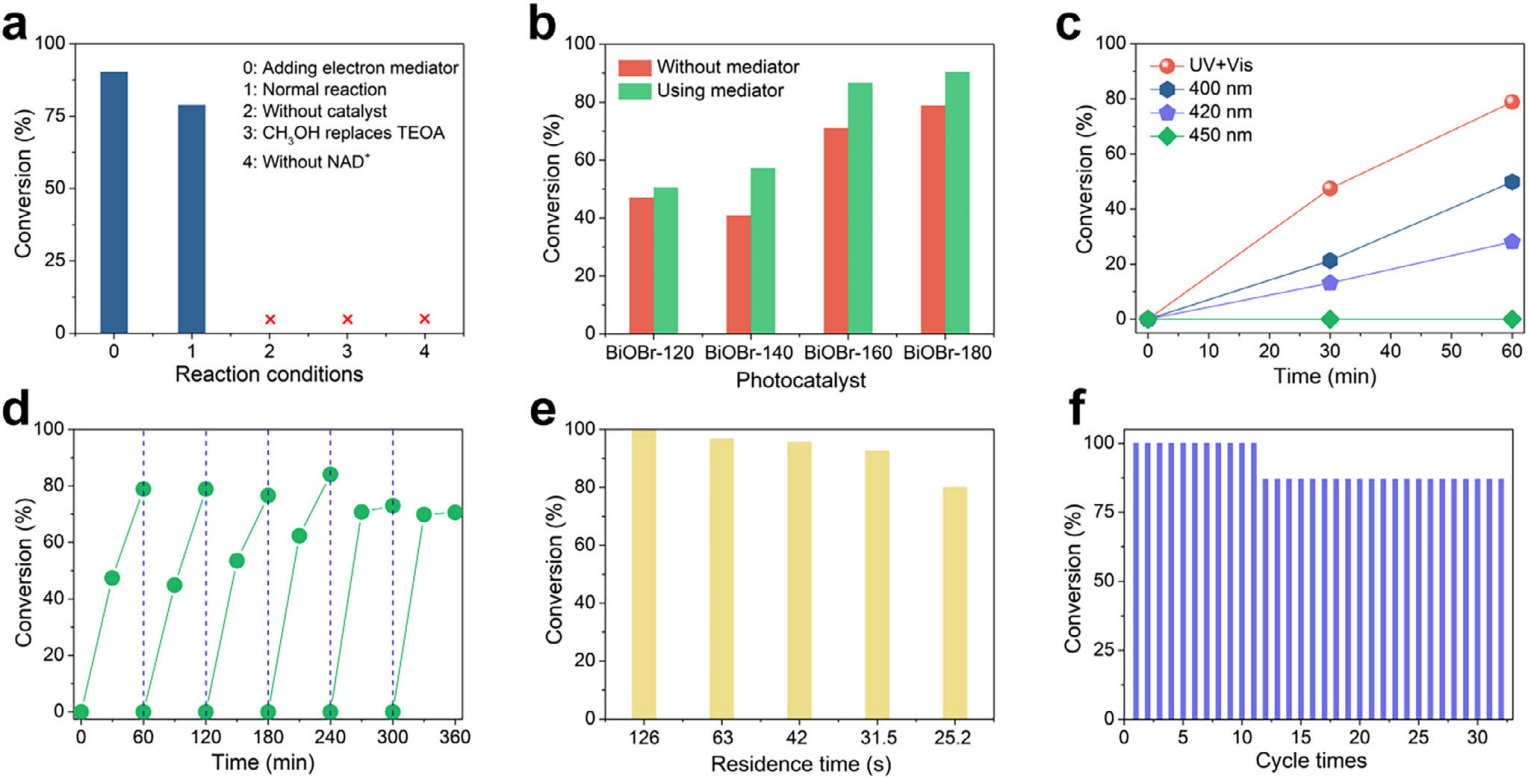
a) Photocatalytic NADH regeneration activity of BiOBr‐180 under different conditions. b) Comparison of NADH regeneration activities over various catalysts. c) Time‐dependent NADH regeneration kinetics of BiOBr‐180 under monochromatic light irradiation. d) Cycling stability of BiOBr‐180 for NADH regeneration over six consecutive runs. e) Microfluidic photocatalytic NADH regeneration activity of BiOBr‐180 at different residence times. f) Operational stability of the microfluidic system for NADH regeneration at a fixed residence time of 126 s.

As a comparison, the photocatalytic activity of BiOBr for coenzyme regeneration in a batch reactor is first investigated. A series of control experiments indicates that NAD^+^, TEOA, and the photocatalyst are essential for the reaction, as the absence of any one component prevents the conversion of NAD^+^ to NADH (**Figure** **3**a). Mechanistically, TEOA serves as a sacrificial electron donor that efficiently quenches photogenerated holes, thereby suppressing charge recombination and sustaining photocatalytic activity. Furthermore, most photocatalytic coenzyme regeneration systems utilize electron mediators containing noble metals, such as [Cp^*^Rh(bpy)H_2_O]^2+^, to achieve efficient NADH generation. The use of electron mediators undoubtedly increases the cost of the catalytic reaction and complicates subsequent separation processes. Therefore, the development of a direct photocatalytic coenzyme regeneration system without the use of electron mediators holds significant research value. Surprisingly, even without electron mediators, the BiOBr photocatalyst efficiently photocatalyzes the conversion of NAD^+^ to NADH, with a conversion of 78.87% (1 h). Under conditions with an electron mediator, the conversion of NAD^+^ to NADH increases to 90.31% (1 h). This conversion exceeds the reported efficiencies of all other photocatalytic coenzyme regeneration systems (Table S1, Supporting Information). ^1^H NMR analysis confirms that the reduction product of NAD^+^ in the absence of electron mediators is physiologically active 1,4‐NADH, with no detectable 1,6‐NADH or 1,2‐NADH (Figure S6, Supporting Information). Quantitative ^1^H NMR further revealed that the BiOBr‐180 photocatalytic NAD^+^ reduction achieved a 57.02% conversion to 1,4‐NADH without electron mediators, demonstrating 72.30% selectivity for 1,4‐NADH formation (Figure S6, Supporting Information). The yield discrepancy between NMR and UV–vis analyses may arise from the formation of an additional product, such as NAD_2_. Figure 3b; Figures S7 and S8 (Supporting Information) display the photocatalytic NADH regeneration performance and UV–vis absorption spectra of various photocatalysts, both with and without electron mediators. The photocatalytic activity increases gradually with the preparation temperature of the catalyst. This is mainly because, as the preparation temperature rises, the thickness size of the BiOBr photocatalyst nanosheets decreases, enhancing charge separation efficiency and improving photocatalytic activity. Additionally, the NADH regeneration activity of BiOBr‐180 under different monochromatic light conditions (without electron mediators) is evaluated (Figure 3c; Figure S9, Supporting Information). The catalytic activity decreases as the irradiation wavelength increases. Under 400 nm monochromatic light, the conversion of NAD^+^ is 49.83% (1 h), while under 420 nm monochromatic light, the conversion decreases to 28.05% (1 h), and the corresponding apparent quantum yields are 1.20% and 0.66%, respectively. A 360 min cycling test demonstrates that the photocatalytic activity of BiOBr‐180 remains stable during cycling, showing excellent cycling stability (Figure 3d; Figure S10, Supporting Information). XPS analysis before and after the reaction indicates that BiOBr‐180 does not undergo significant surface structure changes, further confirming its excellent stability (Figure S11, Supporting Information). Based on these results, we conclude that BiOBr‐180 nanosheets are highly efficient and stable photocatalysts for NADH regeneration, with broad potential for future applications.

Although BiOBr photocatalysts have demonstrated high catalytic activity in batch reaction systems, achieving continuous regeneration of the coenzyme NADH remains a key objective in industrial production. Therefore, the fabricated microfluidic chip is utilized to enable continuous photocatalytic NADH production. As shown in Figure 3e and Figure S12 (Supporting Information), the longer the residence time of the reaction solution within the microfluidic chip, the higher the conversion of NAD^+^. With a residence time of only 126 s, NAD^+^ is fully converted to NADH with a 100% conversion, which, to our knowledge, represents the highest reported conversion efficiency for photocatalytic coenzyme regeneration. Even when the residence time decreases to 25.2 s, the conversion of NAD^+^ remains as high as 79.95%. This outstanding efficiency highlights the advantages of microfluidic chips in photocatalytic coenzyme regeneration reactions. Similarly, we additionally evaluated the performance of our microfluidic system using [Cp^*^Rh(bpy)H_2_O]^2+^ as an electron mediator. The results show that in the presence of the mediator, the system achieved ≈90% conversion of NAD^+^ within a residence time of 126 s. This conversion rate is slightly lower than the nearly quantitative conversion achieved without the mediator, which we speculate may be related to competitive reaction pathways introduced by the mediator. Specifically, in the microfluidic system, the electron mediator may adsorb onto the catalyst surface but fail to desorb effectively due to the absence of agitation or similar mixing mechanisms, thereby hindering subsequent reaction steps (Figure S13, Supporting Information). Furthermore, during a continuous operation test conducted for up to 32 h at a flow rate of 10 µL min^−1^, the NAD^+^ conversion remained at 100% for the first 11 cycles. However, as the test progressed, the conversion gradually decreased to 87%, primarily due to catalyst loss over extended operation (Figure S14, Supporting Information). Nevertheless, the continuous test demonstrated the exceptional stability and catalytic performance of the microfluidic chip in photocatalytic NADH regeneration (Figure 3f; Figure S15, Supporting Information). SEM and XPS analyses of the BiOBr‐180 catalyst before and after the photocatalytic reaction in the microfluidic chip showed no significant changes in morphology or surface structure, confirming its excellent structural stability (Figures S16 and S17, Supporting Information). In summary, the application of microfluidic chips significantly enhances the efficiency and stability of photocatalytic NADH regeneration, providing critical technical support for its industrial‐scale continuous production.

**Figure 4:**
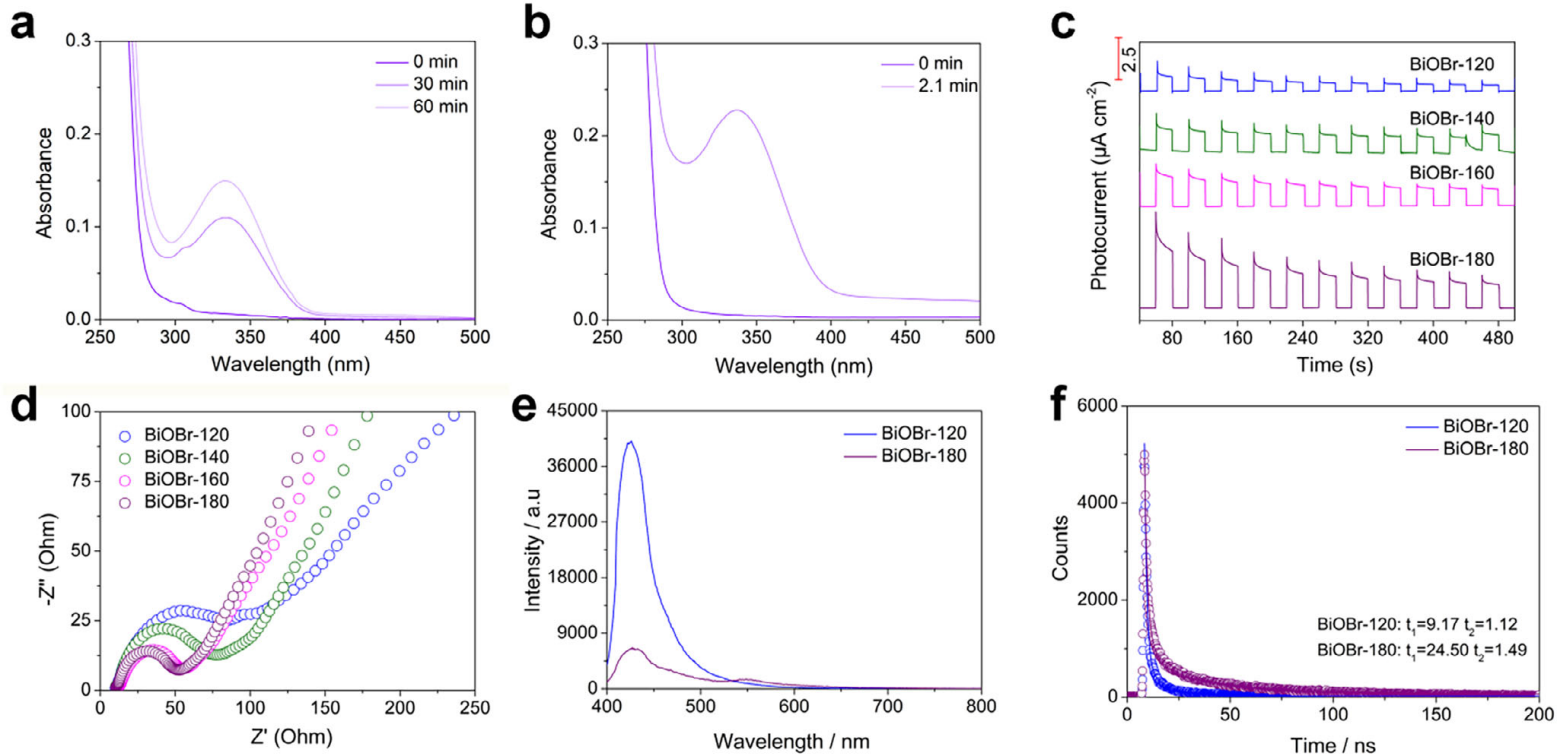
Photocatalytic NADPH regeneration performance of BiOBr‐180: a) Batch reactor test, b) Microfluidic chip test. c) Transient photocurrent response and d) EIS spectra of the series of photocatalysts. e) Steady‐state PL spectra and f) time‐resolved PL spectra of BiOBr‐120 and BiOBr‐180.

Coenzyme NADPH, like NADH, is a crucial cofactor widely involved in cellular energy metabolism and biosynthetic reactions. Compared with batch reactors, microfluidic chips also significantly enhance the efficiency of photocatalytic NADPH regeneration. **Figure** **4**a presents the UV–vis absorption spectrum of NADPH regeneration using BiOBr‐180 nanosheet photocatalysts in a batch reactor without an electron mediator. The characteristic absorption peak of NADPH at 340 nm increases in intensity over time, indicating its formation.^[^ ^27^ ^]^ Within the first 60 min, the absorption peak intensity rises by 0.1428. In contrast, photocatalytic NADPH regeneration using microfluidic chips demonstrates higher efficiency. Without electron mediators, the absorption intensity increases by 0.1985 (Figure 4b). This improvement is primarily due to the advantages of microfluidic chips, including more uniform light distribution, enhanced mass transfer, and precise control of the reaction environment, which collectively lead to a significant enhancement in reaction rate and yield.

In fact, the microfluidic testing method, as an approach for improving catalyst performance, significantly enhances the coenzyme regeneration efficiency. At the same time, the physicochemical properties of the photocatalyst itself are the core factors influencing catalytic activity. Therefore, photoelectric and fluorescence tests are used to evaluate the charge separation efficiency and charge migration rate of the photocatalyst. As the catalyst synthesis temperature increases, the charge separation efficiency and migration rate correspondingly improve, reflected by higher photocurrent density and reduced resonance impedance (Figure 4c,d). The increase in photocurrent density and the gradual reduction in impedance indicate enhanced charge separation efficiency and migration rate, which is consistent with the observed catalytic activity trend. Furthermore, compared to BiOBr‐120, BiOBr‐180 exhibits significantly reduced fluorescence intensity and prolonged fluorescence lifetime, further confirming its superior carrier separation efficiency and high charge migration rate (Figure 4e,f).^[^ ^28^, ^29^, ^30^ ^]^ As the thickness decreases, the migration path of photo‐generated charge carriers becomes shorter, which reduces the resistance faced by carriers during migration, thereby promoting the effective separation of charge carriers and ultimately improving catalytic activity.^[^ ^31^, ^32^ ^]^

**Figure 5:**
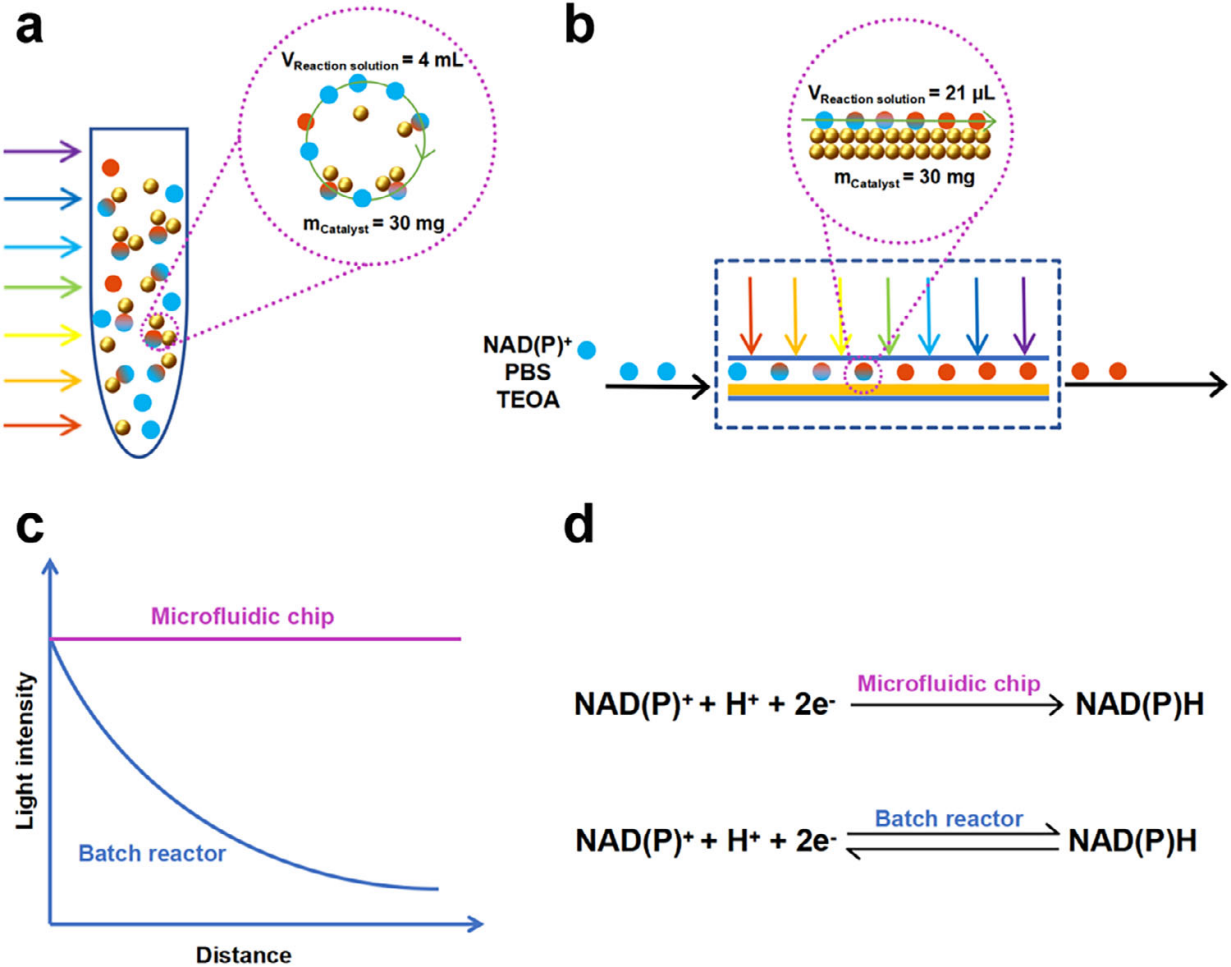
a,b) Schematic illustrations of the intermittent catalytic reaction process and the microfluidic chip‐based catalytic reaction process. c) Variation curve of light intensity as a function of reactor thickness. d) Reaction equation for photocatalytic NAD(P)H regeneration.

In the testing process of photocatalytic coenzyme NAD(P)H regeneration, the microfluidic chip‐based testing method demonstrates significantly higher catalytic activity compared with the conventional batch reactor testing method. This phenomenon can be attributed to the following key factors. First, the high concentration of catalysts significantly enhances reaction efficiency. As shown in **Figure** **5**a, in conventional batch testing, 30 mg of photocatalyst requires mixing with 4 mL of reaction solution, leading to a high degree of catalyst dispersion and limited utilization of active sites. In contrast, in the microfluidic chip‐based testing method, only 21 µL of reaction solution is needed to fully interact with 30 mg of photocatalyst (Figure 5b). The high concentration of catalysts significantly increases the exposure of active sites, thereby greatly improving catalytic efficiency. Second, the uniform distribution of light intensity is another major advantage of microfluidic chips. In batch reactors, as the reactor thickness increases, light intensity gradually attenuates due to the extended optical path, making it difficult to maintain uniform light distribution. This results in reduced reaction efficiency and uneven utilization of catalyst activity (Figure 5c). However, microfluidic chips, with their unique microchannel structure and small reaction scale, enable uniform light distribution, ensuring consistent light intensity on the catalyst surface, thereby further improving reaction efficiency and catalyst utilization. Lastly, the microfluidic chip‐based testing method allows for the immediate separation of reaction products and catalysts, effectively preventing reverse reactions (Figure 5d). This feature is unachievable in conventional batch reactors. In batch reactors, prolonged coexistence of reaction products and catalysts can easily lead to reverse reactions, reducing the yield of target products. In contrast, the continuous flow design of microfluidic chips enables the timely removal of reaction products from the system, significantly enhancing reaction selectivity and yield. In summary, the advantages of the microfluidic chip‐based testing method in terms of catalyst concentration, light uniformity, and product separation efficiency make it exhibit superior catalytic performance in the photocatalytic regeneration of coenzyme NAD(P)H.

## 3 Conclusion

This study successfully establishes a highly efficient, mediator‐free microfluidic platform for photocatalytic regeneration of biologically essential cofactors NAD(P)H, achieved through the strategic integration of ultrathin BiOBr nanosheets as the core photocatalyst. The ingenious architecture of this system facilitates electron‐direct‐proton coupling at the catalyst interface, enabling ultrafast and quantitative NAD^+^ conversion (100%) within a remarkably short timeframe of 126 s. Critically, the platform exhibits outstanding selectivity (72.30%) toward the enzymatically active 1,4‐NADH isomer, a crucial requirement for downstream biocatalytic applications. In addition, the system showed good operational stability, and no serious loss of activity occurred during 32 h continuous operation. This unprecedented combination of rapid kinetics, high selectivity, and long‐term stability under continuous flow conditions represents a significant advancement in photocatalytic cofactor regeneration technology. The work not only sets a new benchmark for the design of integrated photocatalysis systems by elegantly merging microfluidic engineering with tailored nanomaterial science but also powerfully underscores the immense potential of such platforms for driving innovations in sustainable biocatalysis, complex synthetic biology pathways, and next‐generation renewable energy conversion schemes.

## Conflict of Interest

The authors declare no conflict of interest.

## Supporting information



Supporting Information

## Data Availability

The data that support the findings of this study are available from the corresponding author upon reasonable request.
