# Supplementary material for: Electron‐Mediator‐Free Microfluidic Photocatalytic Coenzyme Regeneration with 100% Conversion Efficiency within 126 S
Source: Adv Sci (Weinh). 2025 Nov 7;13(10):e13720. doi: 10.1002/advs.202513720 (PMC12915105; doi:10.1002/advs.202513720)
Supplement: Supplementary file 1 — Supporting Information [file ADVS-13-e13720-s001.docx]

Supporting Information

**Electron-Mediator-Free Microfluidic Photocatalytic Coenzyme Regeneration with 100% Conversion Efficiency within 126 Seconds**

*Yao Chai, Liang Wan, Zirui Pang, Xu Li, Zixuan Jia, Heng Jiang, Chi Chung Tsoi, Huaping Jia, Jinni Shen, Zizhong Zhang, Jinlin Long, Fengjia Xie,** *Yanmei Chen,** *and Xuming Zhang**

**CONTENTS**

Raw Materials.**1**

Synthesis of BiOBr nanosheet photocatalysts.**1**

Synthesis of [Cp^*^Rh(bpy)H_2_O]^2+^ electron mediator.**1**

Preparation of microfluidic chip for regeneration of coenzyme NAD(P)H.**2**

Photocatalytic NAD(P)H regeneration.**2**

Characterization.**3**

Photocurrent and electrochemical impedance measurements.**4**

**Figure S1** SEM images of BiOBr photocatalysts prepared at different temperatures**5**

**Figure S2** TEM images of BiOBr photocatalysts prepared at different temperatures**6**

**Figure S3** AFM images and corresponding heights of a series of photocatalysts prepared under different temperature conditions.**7**

**Figure S4** Theoretical monolayer thickness of BiOBr along the [001] crystal orientation.**8**

**Figure S5** UV-Vis absorption spectra of a series of photocatalysts**9**

**Figure S6** ^1^H NMR spectra of the solution before and after the reaction**10**

**Figure S7** Time-resolved UV-visible absorption spectra of a series of BiOBr photocatalysts for photocatalytic regeneration of coenzyme NADH with the assistance of electron mediators**12**

**Figure S8** Time-resolved UV-visible absorption spectra of a series of BiOBr photocatalysts for photocatalytic regeneration of coenzyme NADH in the absence of electron mediators**13**

**Figure S9** Time-resolved UV-visible absorption spectra of coenzyme NADH regeneration by BiOBr-180 under different monochromatic light without an electron mediator**14**

**Figure S10** Time-resolved UV-visible absorption spectra of six cycles of photocatalytic NADH regeneration on BiOBr-180.**15**

**Figure S11** XPS spectra of the catalyst before and after the reaction**16**

**Figure S12** Time-resolved UV-visible absorption spectra of photocatalytic NADH regeneration by BiOBr-180 at different residence times in a microfluidic chip.**17**

**Figure S13** Photocatalytic NADH regeneration in a microfluidic chip with an electron mediator.**18**

**Figure S14** Photographs of the microfluidic chip pre- and post-reaction: (a) initial state and (b) after operation**90**

**Figure S15** Time-resolved UV-visible absorption spectroscopy for stability testing of BiOBr-180 in microfluidic chips**20**

**Figure S16** Post-reaction SEM of the photocatalyst deposited in the microfluidic chip**21**

**Figure S17** Comparison of the XPS spectra for the catalyst in the microfluidic chip pre- and post-reaction.**22**

**Figure S18** NADH calibration curve**23**

**Table S1.** A comparative assessment of photocatalytic NADH regeneration efficiency was conducted between a microfluidic-based reaction system and conventional batch reactors**24**

**Supporting References26**

Raw Materials.

Hexadecyltrimethylammonium bromide [CH_3_(CH_2_)_15_N(Br)(CH_3_)_3_, ≥98%], bismuth(III) nitrate pentahydrate [Bi(NO_3_)_3_·5H_2_O, ACS reagent, ≥98.0%], sodium bromide [NaBr, ACS reagent, ≥99.0%], β-nicotinamide adenine dinucleotide hydrate (NAD^+^, ≥99%), β-nicotinamide adenine dinucleotide, reduced disodium salt hydrate (β-NADH, ≥97%), β-nicotinamide adenine dinucleotide phosphate hydrate (β-NADP^+^, ≥95%), pentamethylcyclopentadienylrhodium(III) chloride dimer [(Cp*RhCl_2_)_2_, ≥97%], triethanolamine (TEOA, Vetec™, reagent grade, 97%), methanol (≥99.8%, ACS reagent, suitable for EPA 1613), and 2,2’-bipyridyl (C_10_H_8_N_2_, ≥99%) are purchased from Sigma-Aldrich Chemical Reagent Co., Ltd. All chemical reagents are used directly without any treatment.

Synthesis of BiOBr nanosheet photocatalysts.

BiOBr nanosheet photocatalysts were synthesized via a one-step hydrothermal method. Initially, 0.5 g of CTAB was dissolved in 80 mL of deionized water under stirring. Subsequently, 2 mmol of Bi(NO_3_)_3_·5H_2_O was added to the solution and stirred until fully dispersed. Next, 4 mmol of NaBr was introduced and stirred until completely dissolved. The resulting mixture was continuously stirred for 1 hour before being transferred into an 80 mL polytetrafluoroethylene-lined autoclave, which was then sealed within a stainless steel reactor for the hydrothermal reaction. The reaction was carried out at 180 °C for 24 hours. Upon completion, the solid product was collected by filtration, washed thoroughly with deionized water, and dried in a vacuum oven at 60 °C. To investigate the effect of reaction temperature, BiOBr nanosheets were synthesized at 120, 140, 160, and 180 °C under identical conditions. The resulting samples were designated as BiOBr-120, BiOBr-140, BiOBr-160, and BiOBr-180, respectively.

Synthesis of [Cp^*^Rh(bpy)H_2_O]^2+^ electron mediator.

The synthesis method of [Cp^*^Rh(bpy)H_2_O]^2+^ adheres to a previously reported procedure. Initially, 0.1 mmol of (Cp^*^RhCl_2_)_2_ and 0.2 mmol of 2,2'-bipyridine are added to 4 mL of methanol and stirred until dissolved. Once the solution naturally evaporates to about 1 mL, 40 mL of cold ether is quickly added to precipitate [Cp^*^Rh(bpy)Cl]Cl. After filtering the ether, 8 mL of deionized water is added to obtain [Cp^*^Rh(bpy)H_2_O]^2+^. The [Cp^*^Rh(bpy)H_2_O]^2+^ solution is then stored at 8°C in the dark for future use as an electron mediator in the photocatalytic regeneration of NAD(P)H.

Preparation of microfluidic chip for regeneration of coenzyme NAD(P)H.

The microfluidic chip was fabricated using standard photolithography, as detailed below. Initially, SU8 2050 photoresist was applied to a silicon wafer and spun at 3000 rpm for 30 s using a spin coater. Pre-baking was then performed at 65 °C for 3 min, followed by 95 °C for 6.5 min. The wafer was subsequently exposed under a photolithography system for 22.89 s. After exposure, post-baking was conducted at 65 °C for 1 min and 95 °C for 6 min. The wafer was then developed in a developer solution for 5 min and rinsed with isopropanol (IPA) to obtain the desired channel structures. Next, polydimethylsiloxane (PDMS) was poured onto the silicon wafer. After curing at 85°C for 1 hour, the PDMS layer was peeled off and plasma-bonded to a glass slide coated with the catalyst, completing the fabrication of the microfluidic chip. The reaction chamber of the chip measured 2.0 cm × 1.5 cm × 70 μm. The catalyst was loaded onto the glass slide using a brushing method. A total of 30 mg of catalyst was dispersed in 1 mL of ethanol and agitated for 20 min using a vortex mixer to ensure uniform dispersion. The catalyst suspension was then brushed onto the glass slide, ensuring that the coated area matched the reaction chamber dimensions (2.0 cm × 1.5 cm × 70 μm). The catalyst was immobilized within the predefined reaction area via a slot-die coating process. The specific steps were as follows: First, the catalyst powder (~30 mg) was dispersed in 0.5 mL of ethanol and sonicated for 30 minutes to form a uniform slurry. Subsequently, the slurry was precisely coated onto the substrate using an automated coating machine. Finally, the coated substrate was dried at 60 °C for 6 hours to remove the solvent, ensuring the catalyst was firmly adhered.

Photocatalytic NAD(P)H regeneration.

Photocatalytic NAD(P)H Regeneration Procedure: A 30 mL quartz tube served as the photoreactor. The reaction mixture contained 20 mg of photocatalyst, 1 mL of NAD(P)^+^ solution (1 mM), 1 mL of triethanolamine (TEOA) solution (15.0 wt%), and 2 mL of phosphate buffer (pH 7.4). Prior to illumination, the suspension was stirred in the dark for 30 minutes to establish adsorption-desorption equilibrium. Irradiation was performed using a 300 W xenon lamp (PLS-SXE300E, light intensity 1.34 mW cm^-2^ at 15 cm distance) simulating solar spectrum. Aliquots (1 mL) were withdrawn every 30 minutes during illumination. Each aliquot was immediately filtered through a syringe membrane filter (0.22 μm) to remove catalyst particles. NAD(P)H concentration in the filtrate was quantified by UV-Vis spectroscopy (PerkinElmer, λ = 250–800 nm, scan rate 8 nm s^-1^) using an external calibration curve (Figure S18). For specific quantification of enzymatically active 1,4-NADH, deuterated water (D₂O) was employed as the solvent. Post-reaction solutions were analyzed by ^1^H NMR spectroscopy (Jeol ECZ500R). The characteristic 1,4-NADH proton signal (δ = 6.8 ppm), well-resolved in D₂O without interference from the reaction matrix, was integrated for quantification. When electron mediators were employed, 50 μL of mediator solution was added to the initial reaction mixture. NADH regeneration yield and selectivity were calculated according to Equations 1 and 2. Apparent quantum yield (AQY) was determined using Equations 3 and 4.

$$Selectivity (\%) = \frac{1,4-NADH yield}{Total yield} \times100\% (2)$$

I: Light intensity (W m^-2^), S: Illumination area S ≈ 0.015 × 0.01 (m^-2^), λ: Monochromatic light wavelength (m), t: Light time (s), h: Planck's constant (6.626 × 10^-34^), c: Speed of light (3 × 10^8^ m s^-1^).

To test photocatalytic coenzyme regeneration using the microfluidic chip, 1 mL of NAD(P)^+^ solution (1 mM), 1 mL of TEOA solution (15.0 w/v%), and 2 mL of buffer solution (pH 7.4) were placed in a syringe. Using a syringe pump, the solution was injected into the microfluidic chip at varying flow rates. The reaction solution was collected at the outlet of the chip for analysis.

Characterization.

The crystal structure of the catalyst was characterized by X-ray powder diffraction (XRD) using a Rigaku SmartLab diffractometer. The micromorphology and crystal structure were further examined by transmission electron microscopy (TEM) on an FEI Tecnai T12 microscope. Surface chemical composition was analyzed via X-ray photoelectron spectroscopy (XPS) on a Thermo ESCALAB 250XI instrument, with binding energies referenced to the C 1s peak (284.8 eV). Ultraviolet-visible diffuse reflectance spectra (UV-vis DRS) were recorded using a PerkinElmer UV-vis-NIR spectrometer equipped with an integrating sphere. Photoluminescence (PL) and time-resolved photoluminescence (TRPL) spectra were acquired on an Edinburgh Instruments FLS1000 spectrometer. Monochromatic light intensities (400 nm: 4.6 mW cm^-2^; 420 nm: 4.8 mW cm^-2^) were determined using a calibrated silicon photodiode coupled to an ELITETECH solar simulator.

Photocurrent and electrochemical impedance measurements.

The CHI660 electrochemical workstation is used for photoelectric performance testing. The working electrode is prepared as follows: 10 mg of the photocatalyst is dispersed in 1 mL of ethanol, 20 μL of 5% Nafion solution is added, and ultrasonic dispersion is performed. 20 μL of the resulting dispersion is dropped onto a 1 cm^2^ conductive glass surface and air-dried. For photocurrent measurement, a Pt plate is used as the counter electrode, Ag/AgCl is used as the reference electrode, and a 0.2 M Na_2_SO_4_ solution is used as the electrolyte. For electrochemical impedance measurement, the open circuit voltage is used as the initial voltage, and the frequency range is 0.01 Hz to 100 kHz. A 0.5 M KCl solution and a 5.0 mM K_3_[Fe(CN)_6_]/K_4_[Fe(CN)_6_] solution are used as electrolytes.

**
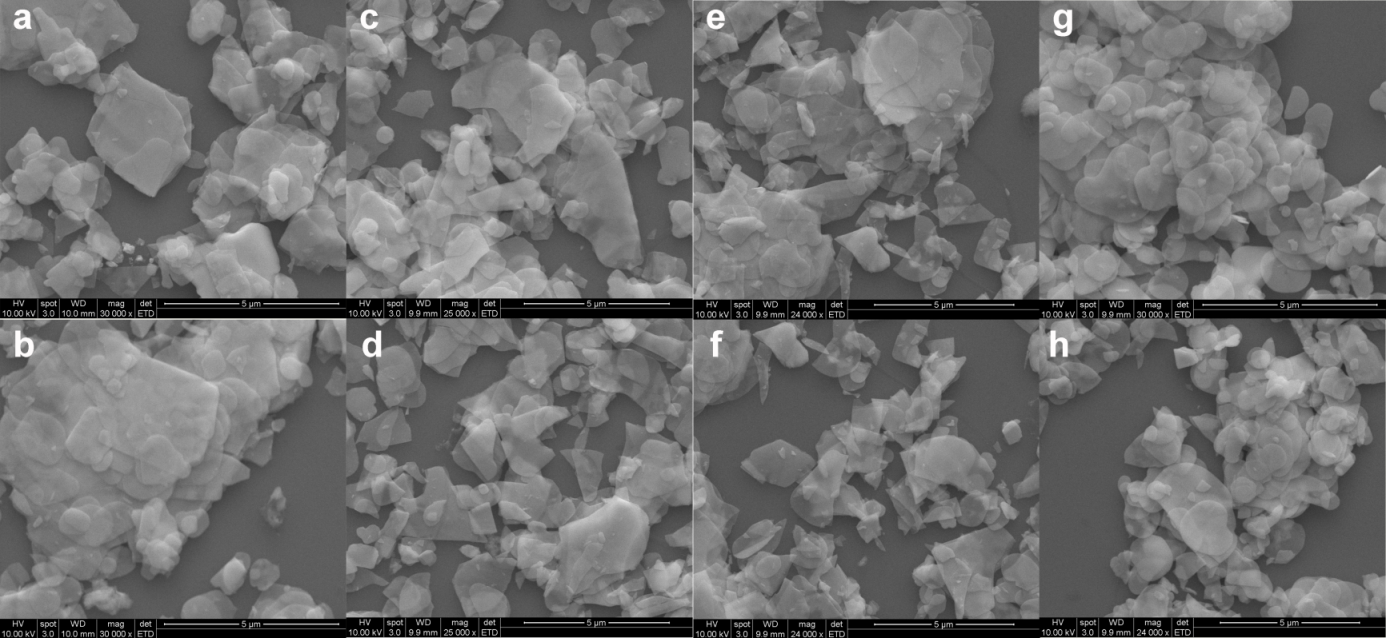
**

**Figure S1.** SEM images of BiOBr photocatalysts prepared at different temperatures. (a, b) BiOBr-120, (c, d) BiOBr-140, (e, f) BiOBr-160, and (g, h) BiOBr-180.


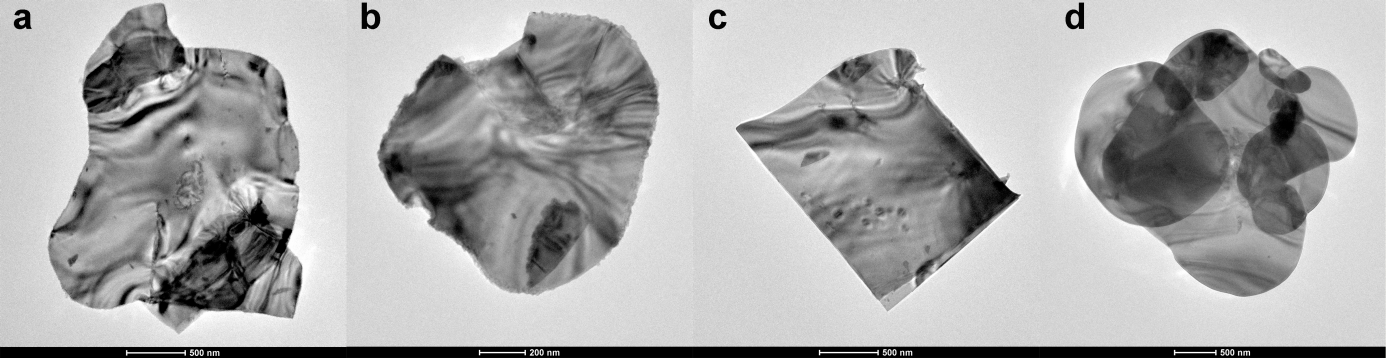


**Figure S2.** TEM images of BiOBr photocatalysts prepared at different temperatures. (a) BiOBr-120, (b) BiOBr-140, (c) BiOBr-160, and (d) BiOBr-180.


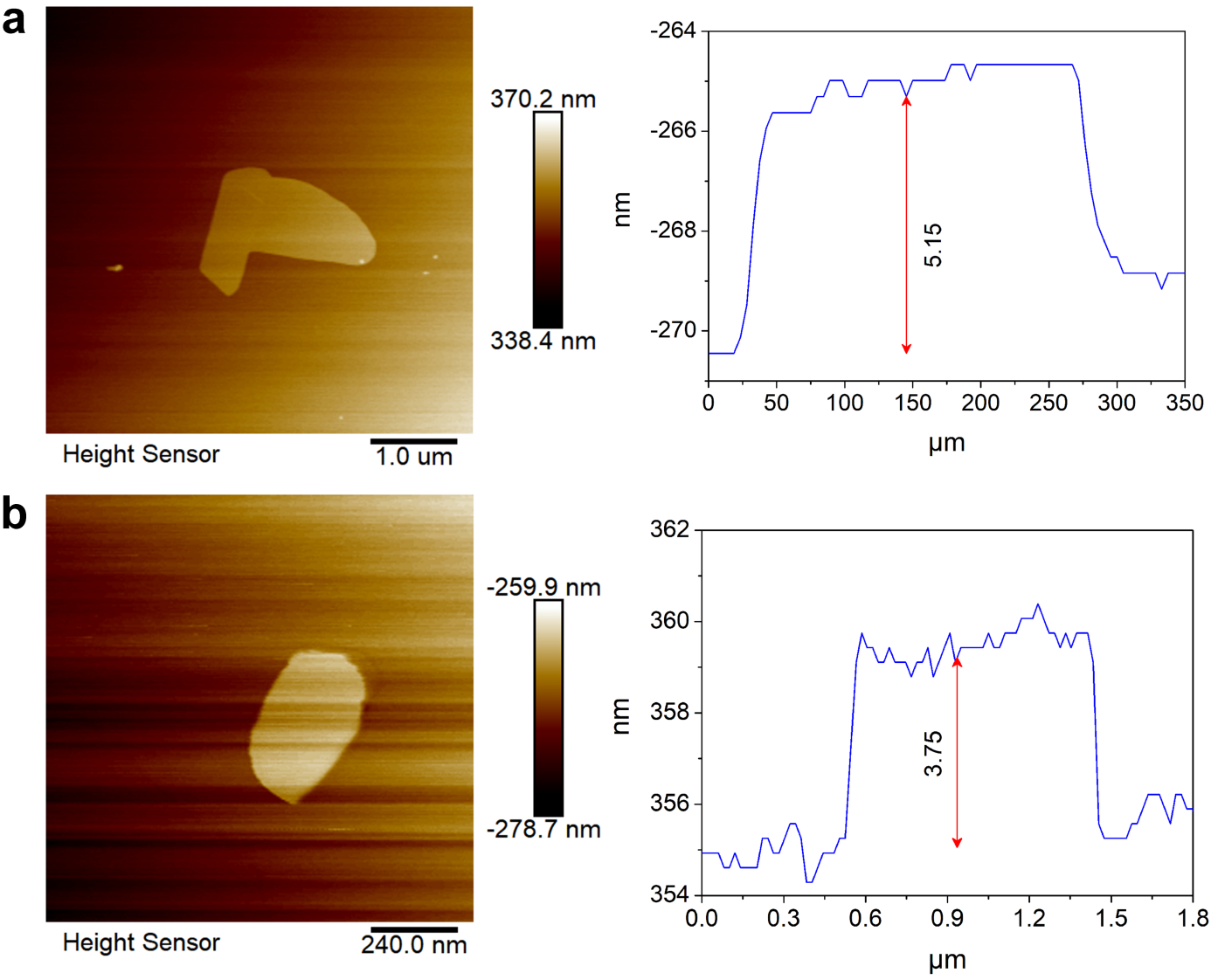


**Figure S3.** AFM images and corresponding heights of a series of photocatalysts prepared under different temperature conditions. (a) BiOBr-140, (b) BiOBr-160.


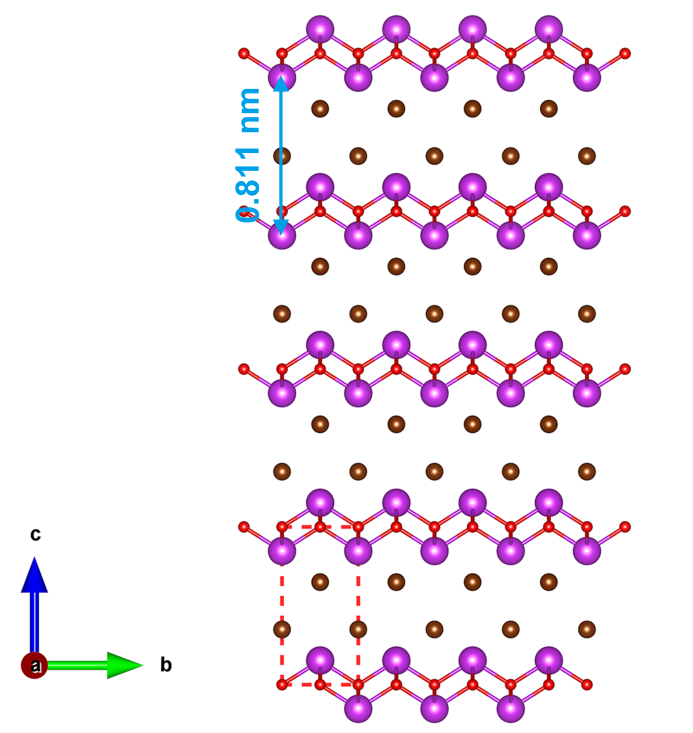


**Figure S4.** Theoretical monolayer thickness of BiOBr along the [001] crystal orientation.

The theoretical monolayer thickness of BiOBr along the [001] crystal orientation is about 0.811 nm (half of the unit cell parameter along the c-axis, c/2 = 0.811 nm).


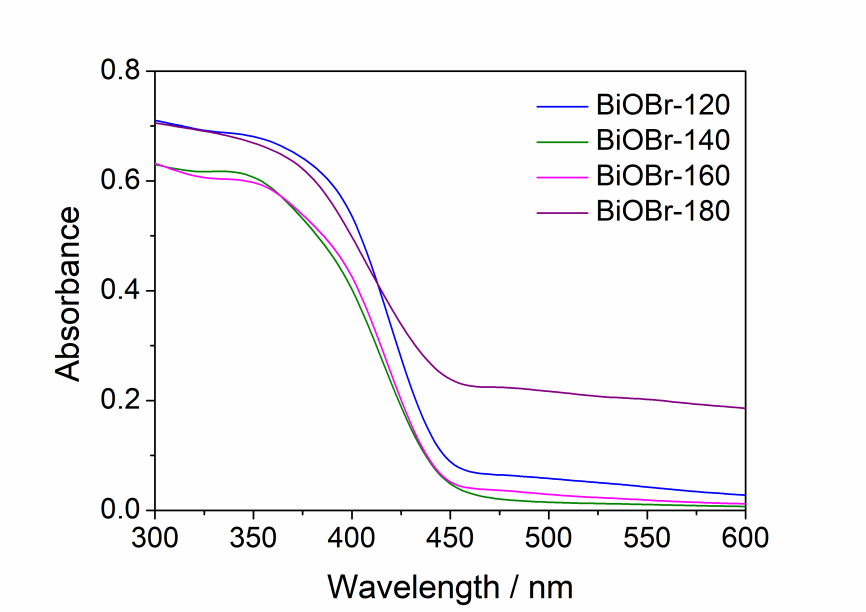


**Figure S5.** UV-Vis absorption spectra of a series of photocatalysts.

**
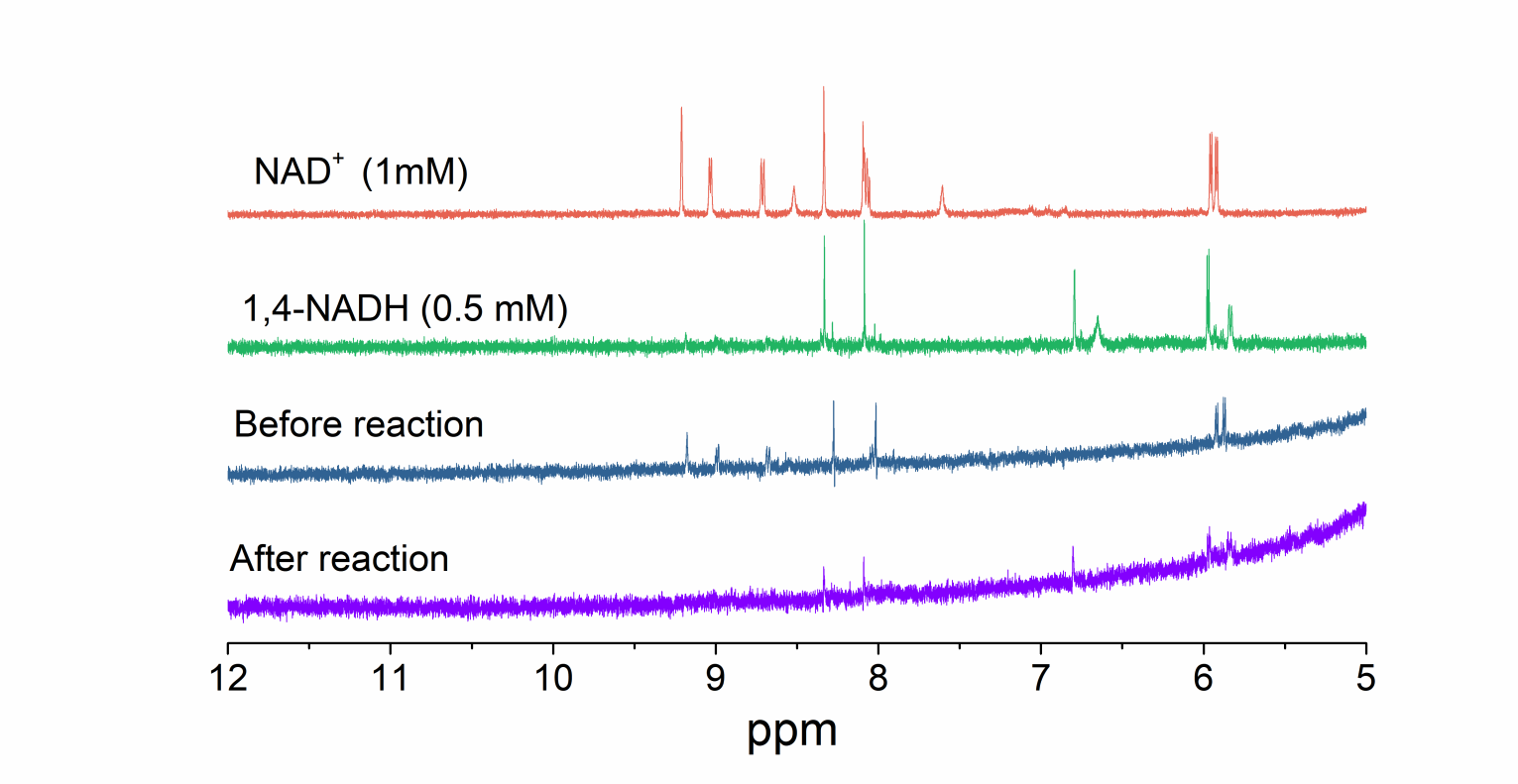
**

**Figure S6.** ^1^H NMR spectra of the solution before and after the reaction.

To further characterize the NAD^+^ reduction products, we conducted ^1^H NMR analysis to monitor chemical shift changes before and after the reaction. The pre-reaction solution exhibited a characteristic NAD^+^ signal at 9.27 ppm,^[1]^ which disappeared upon reaction completion and was replaced by a new peak at 6.87 ppm, corresponding to 1,4-NADH.^[1]^ Notably, no signals attributable to 1,6-NADH (~7.02 ppm) or 1,2-NADH (~6.63 and ~7.38 ppm) were observed in the post-reaction spectrum. For quantitative analysis of 1,4-NADH, deuterated water was employed as the solvent, and the post-reaction solution was analyzed using a Jeol ECZ500R NMR spectrometer. The quantification relied on the integrated peak area of the well-resolved 1,4-NADH proton signal (δ = 6.8 ppm), which was free from interference by the reaction system. Quantitative ^1^H NMR revealed that the BiOBr-180 photocatalytic NAD^+^ reduction achieved 57.02% conversion to 1,4-NADH in the absence of an electron mediator, with 72.30% selectivity for 1,4-NADH formation. The discrepancy between NMR and UV-vis yields may stem from the formation of side products such as NAD_2_.

Standard Calibration: A standard solution of authentic 1,4-NADH with a known concentration (C_1_) was analyzed to measure the integrated peak area (A_1_) at δ = 6.8 ppm.

Sample Analysis: The post-reaction solution was analyzed under the same operational conditions to measure the corresponding peak area (A_2_).

Concentration Calculation: The concentration of 1,4-NADH in the reaction mixture (C_2_) was determined using the following relationship:

C_2_ = (A_2_ / A_1_) × C_1_

The moles of 1,4-NADH produced were calculated from its concentration (C_2_) and the reaction volume (V = 4 mL). These values were then used to determine the key metrics as follows:

Moles of 1,4-NADH produced = C_2_ × V = 0.14255 mM × 4 mL × 10-3 = 0.5702 × 10^-3^ mmol

Conversion of NAD^+^: This was calculated based on the initial amount of NAD^+^.

Initial moles of NAD^+^ = 1 mM × 1 mL × 10^-3^ = 1.0 mmol× 10^-3^

Conversion (%) = (Moles of 1,4-NADH produced / Initial moles of NAD^+^) × 100% = (0.5702 × 10^-3^ / 1.0 × 10^-3^) × 100% = 57.02%

Selectivity (%) = (Moles of 1,4-NADH produced) / (Moles of NAD^+^ consumed) × 100% = (0.5702 × 10^-3^ / 0.7230 × 10^-3^) × 100% = 78.87%


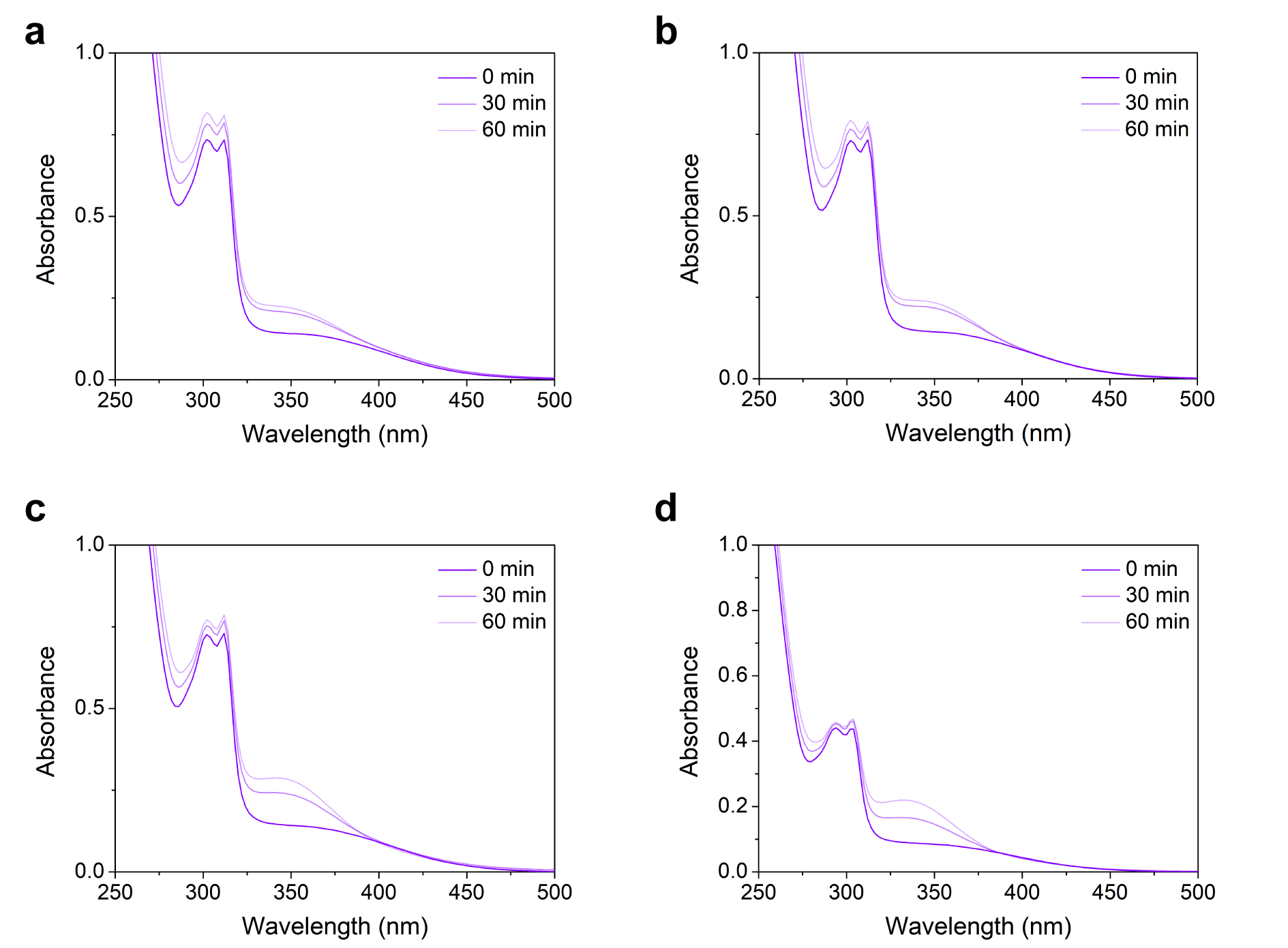


**Figure S7.** **Time-resolved UV-visible absorption spectra of a series of BiOBr photocatalysts for photocatalytic regeneration of coenzyme NADH with the assistance of electron mediators.** (a) BiOBr-120, (b) BiOBr-140, (c) BiOBr-160, and (d) BiOBr-180.


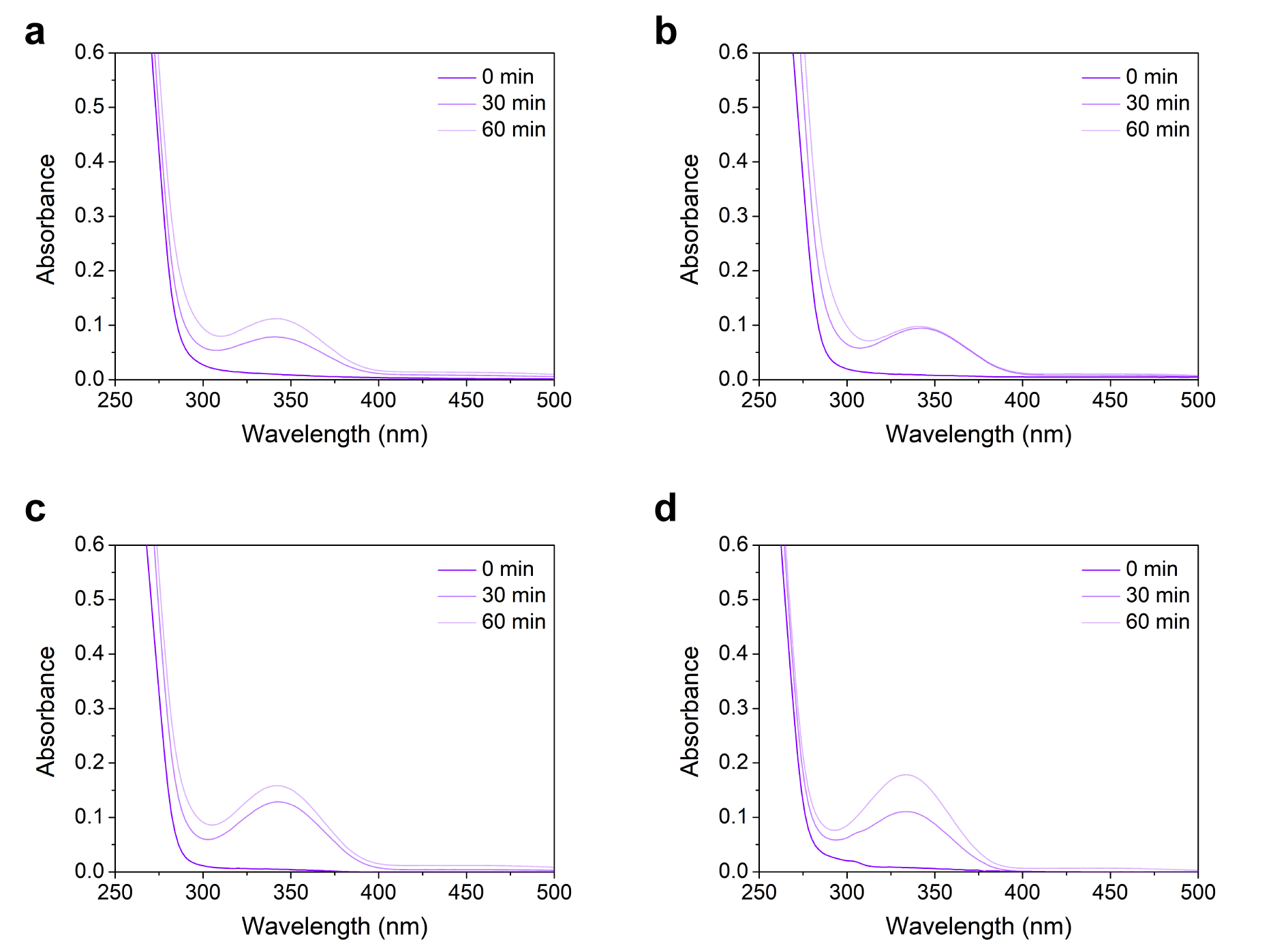


**Figure S8. Time-resolved UV-visible absorption spectra of a series of BiOBr photocatalysts for photocatalytic regeneration of coenzyme NADH in the absence of electron mediators.** (a) BiOBr-120, (b) BiOBr-140, (c) BiOBr-160, and (d) BiOBr-180.


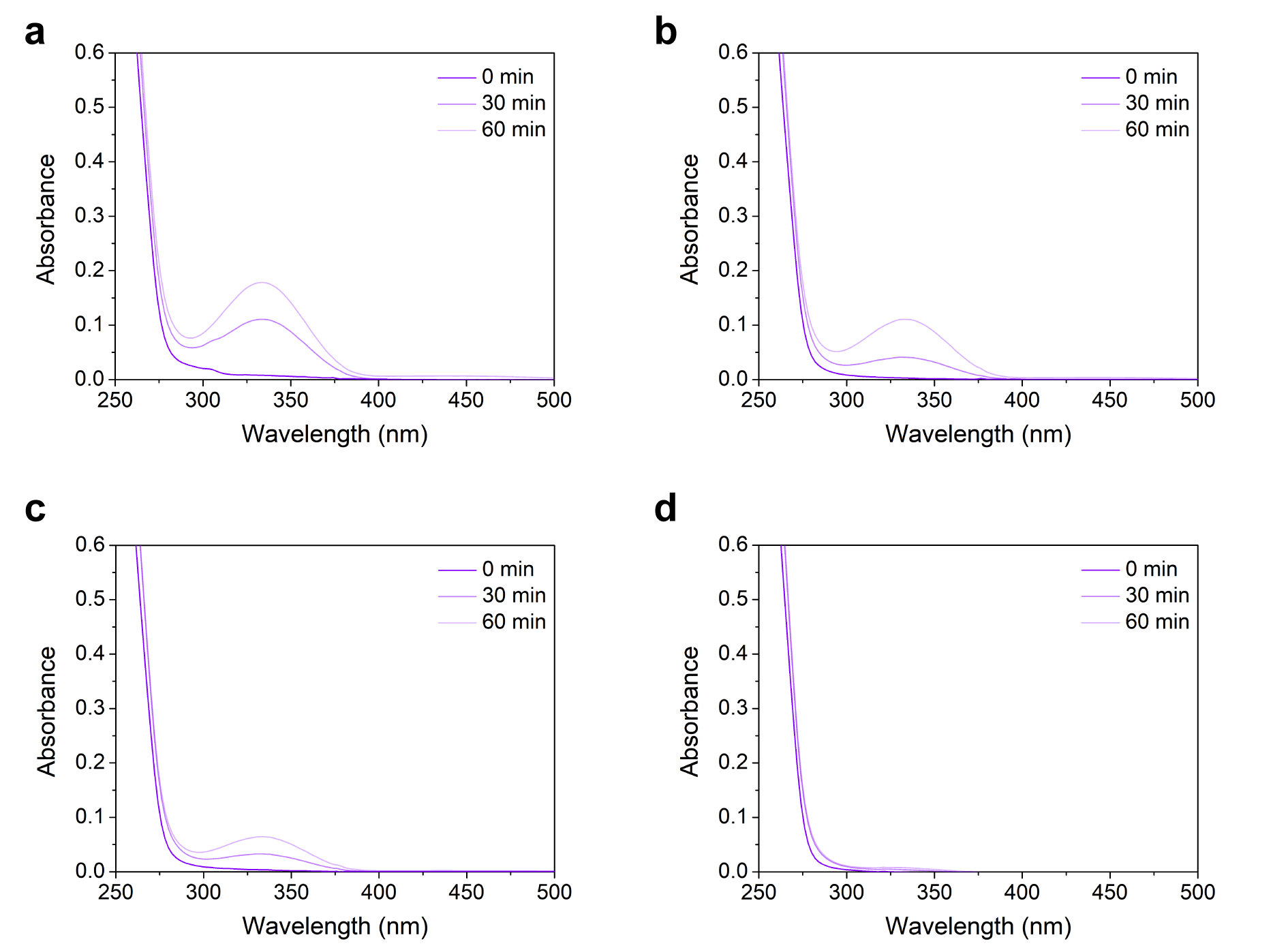


**Figure S9. Time-resolved UV-visible absorption spectra of coenzyme NADH regeneration by BiOBr-180 under different monochromatic light without an electron mediator.** (a) UV+Vis, (b) 400 nm, (c) 420 nm, and (d) 450 nm.


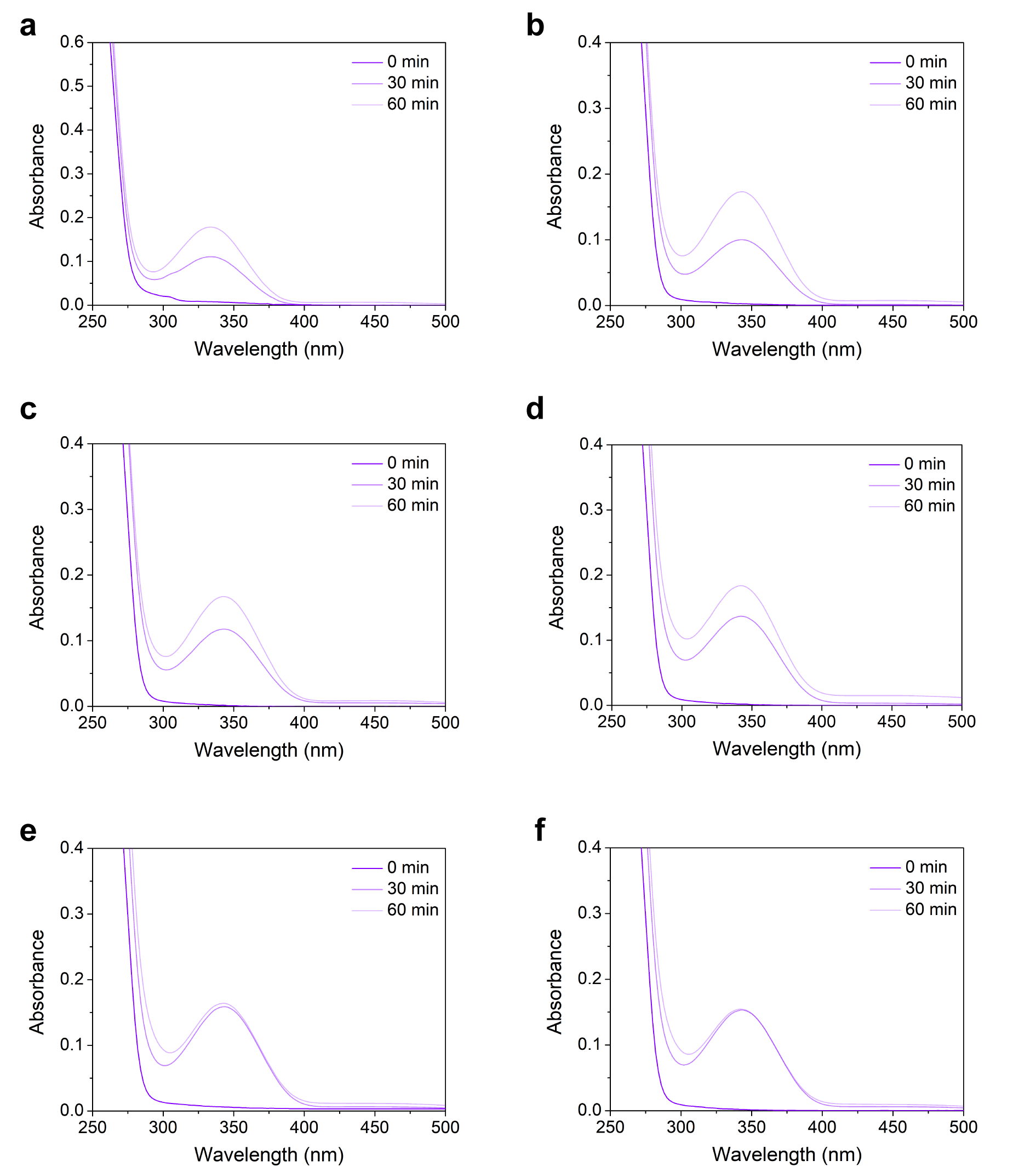


**Figure S10. Time-resolved UV-visible absorption spectra of six cycles of photocatalytic NADH regeneration on BiOBr-180.** (a) First cycle, (b) Second cycle, (c) Third cycle, (d) Fourth cycle, (e) Fifth cycle, and (f) Sixth cycle.


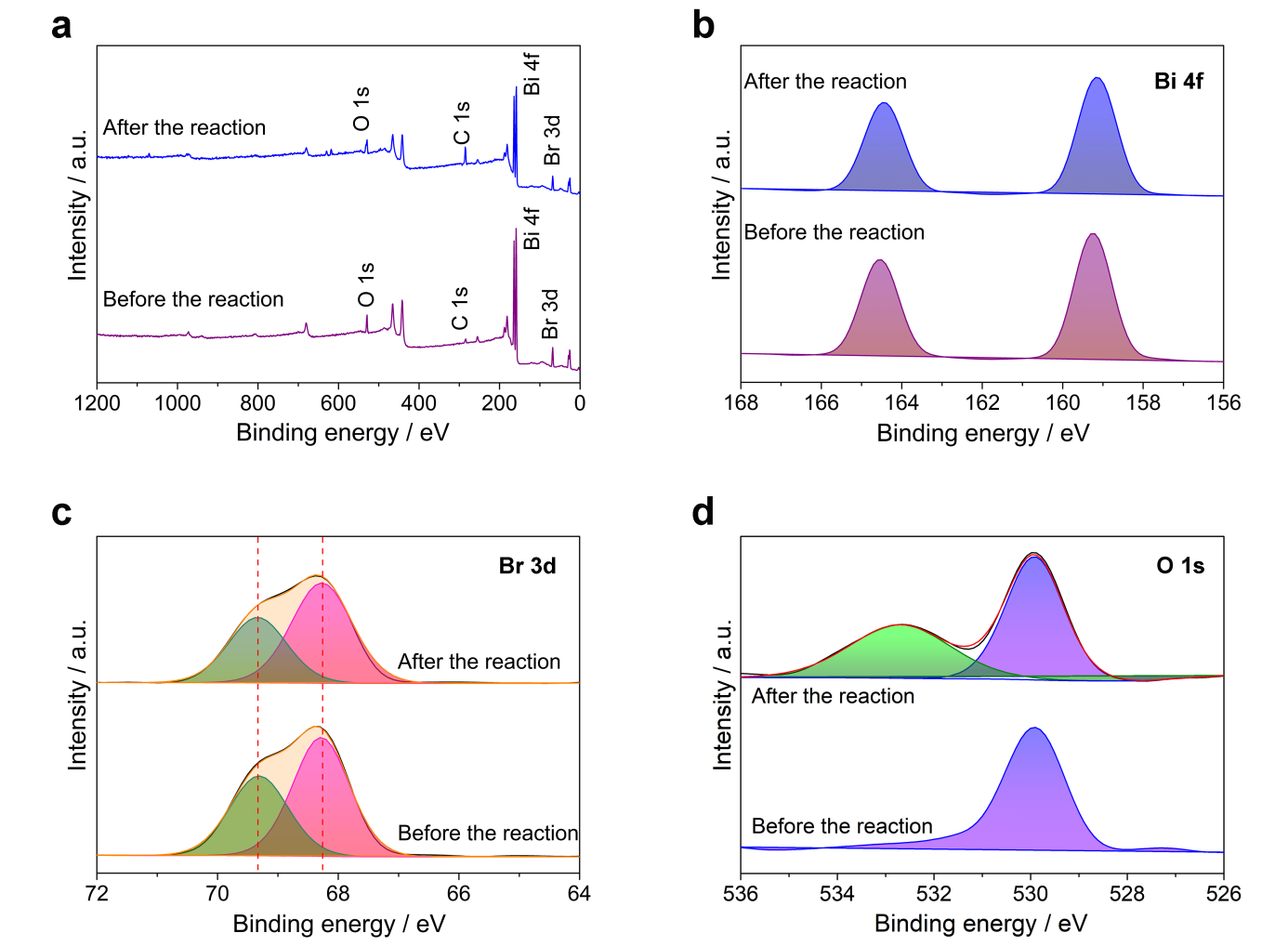


**Figure S11**. **XPS spectra of the catalyst before and after the reaction.** (a) Full spectrum, (b) Bi 4f, (c) Br 3d, and (d) O 1s.

The structural integrity of the catalyst before and after cyclic reactions was investigated via X-ray photoelectron spectroscopy. As illustrated in Figure S11a, the catalyst retained its primary elemental composition (Bi, O, Br) throughout the reaction process. The Bi 4f and Br 3d spectra exhibited minimal variation between the pristine and post-reaction states, indicating no significant chemical alterations in these elements (Figure S11b and Figure S11c). Notably, the O 1s spectrum underwent substantial modification after the reaction, with the emergence of a distinct peak at 532.71 eV (Figure S11d). This new feature corresponds to the C–O bond of organic species, likely originating from the surface adsorption of NADH or TEOA during catalysis. Collectively, the XPS data demonstrate preserved elemental composition and structural consistency of the catalyst post-reaction, confirming its robust stability under operational conditions.


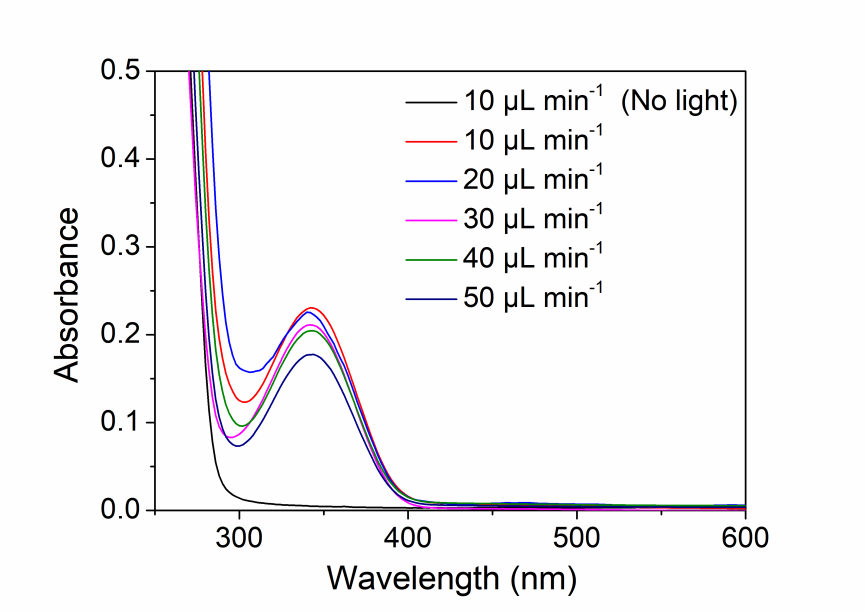


**Figure S12**. Time-resolved UV-visible absorption spectra of photocatalytic NADH regeneration by BiOBr-180 at different residence times in a microfluidic chip.





**Figure S13**. Photocatalytic NADH regeneration in a microfluidic chip with an electron mediator.


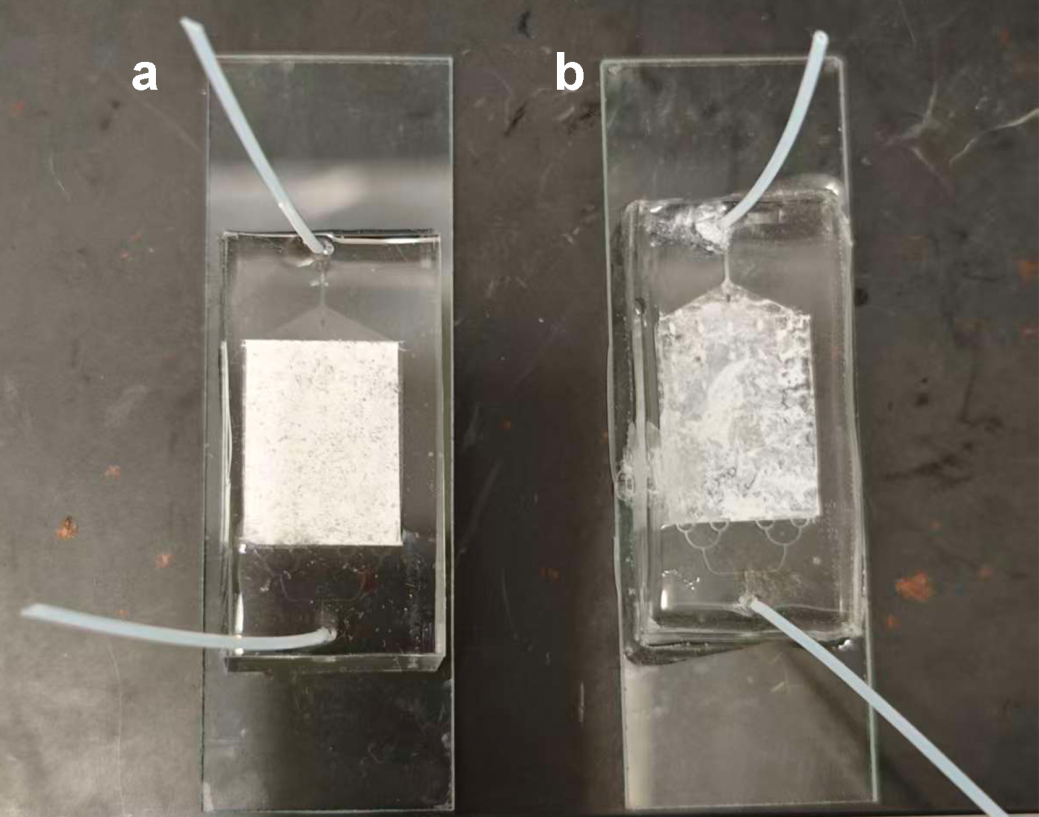


**Figure S14:** Photographs of the microfluidic chip pre- and post-reaction: (a) initial state and (b) after operation.


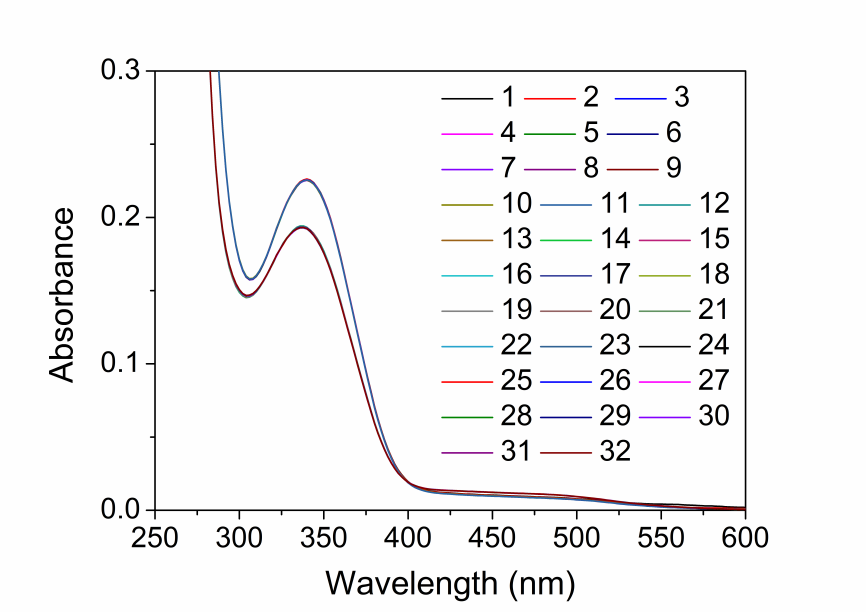


**Figure S15**. Time-resolved UV-visible absorption spectroscopy for stability testing of BiOBr-180 in microfluidic chips.


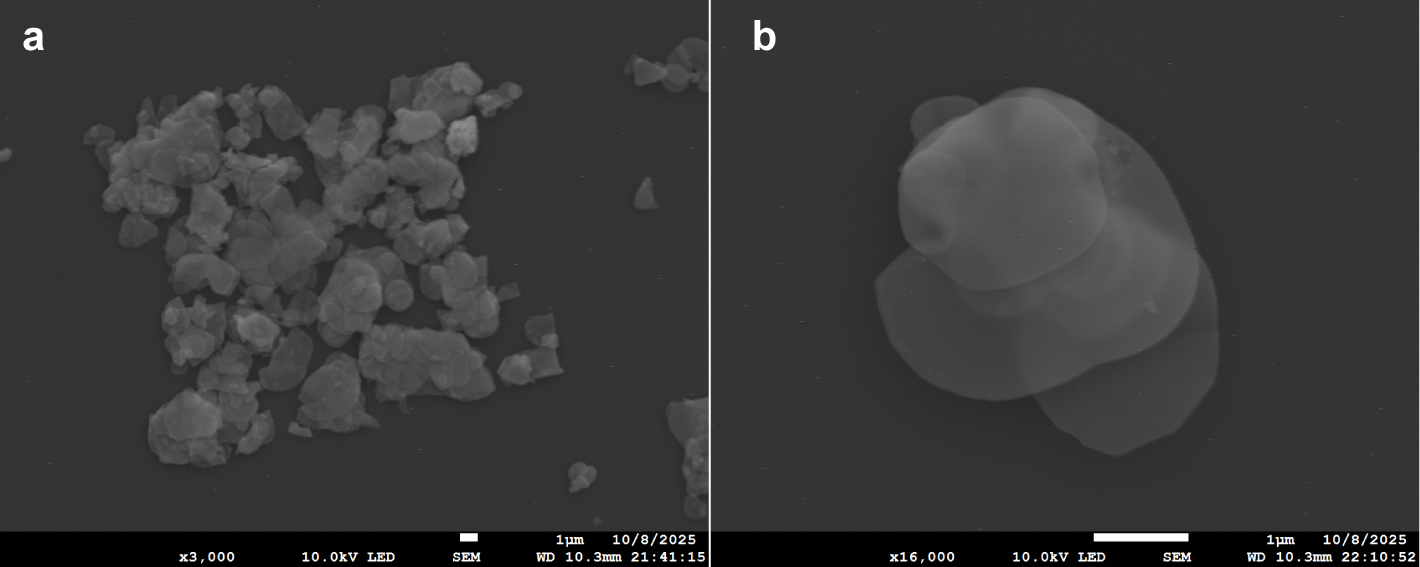


**Figure S16.** Post-reaction SEM morphology of the photocatalyst deposited in the microfluidic chip.


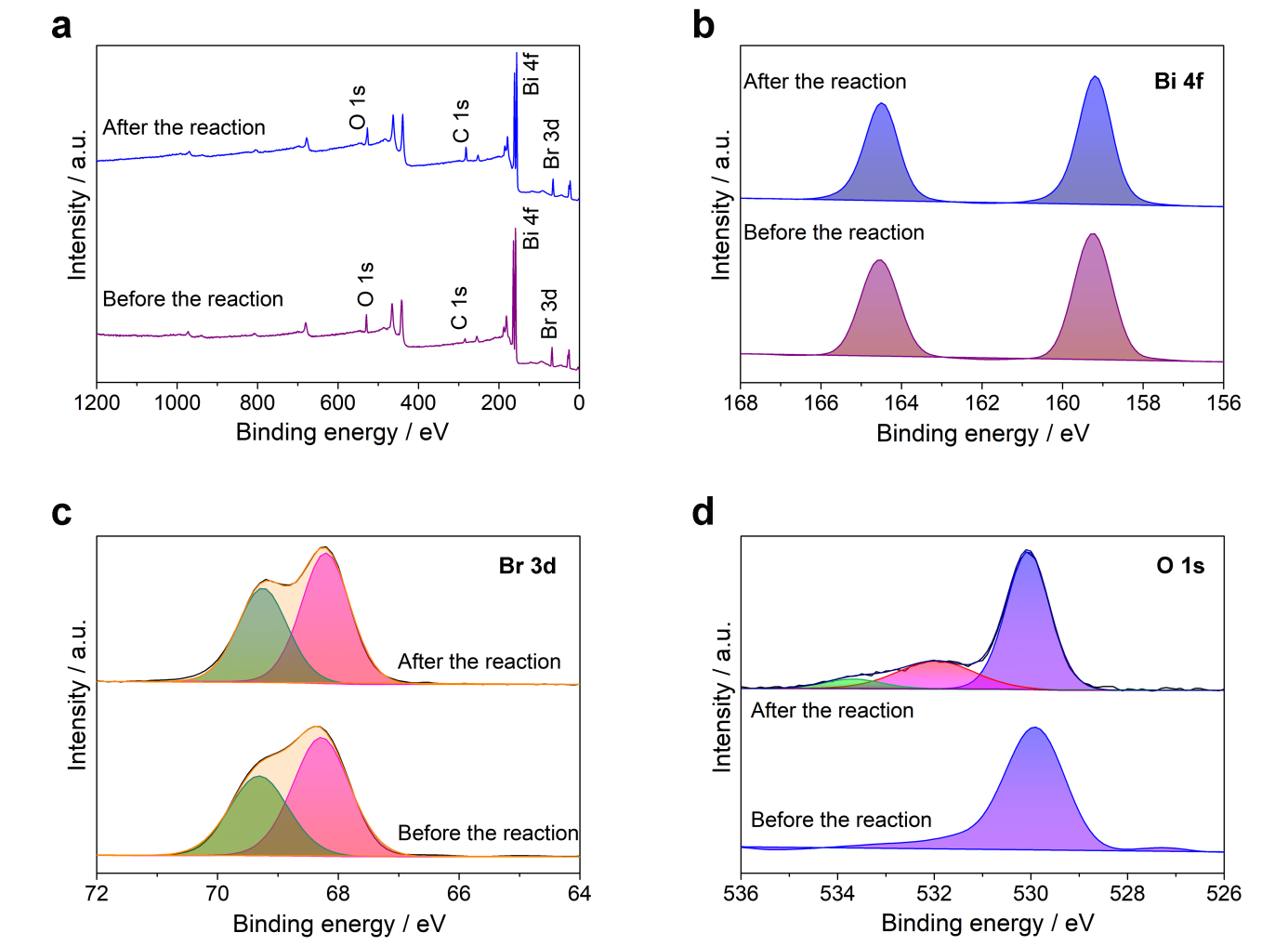


**Figure S17.** Comparison of the XPS spectra for the catalyst in the microfluidic chip pre- and post-reaction: (a) full spectrum, (b) Bi 4f, (c) Br 3d, and (d) O 1s core-level spectra.

XPS analysis reveals that the binding energies and chemical states of Bi and Br remain virtually unchanged compared to the fresh catalyst, indicating the robust stability of the catalyst's core structure. The noticeable change observed in the O 1s spectrum is indeed insightful. The two new peaks at 532.00 eV and 533.72 eV, which emerged after the reaction, are typically attributed to surface-adsorbed oxygen species, such as hydroxyl groups (-OH) or water molecules (H_2_O). This change does not indicate degradation of the BiOBr lattice itself, but rather reflects the interaction between the catalyst surface and the reaction environment, which is a common and expected phenomenon for surface-mediated catalytic processes.


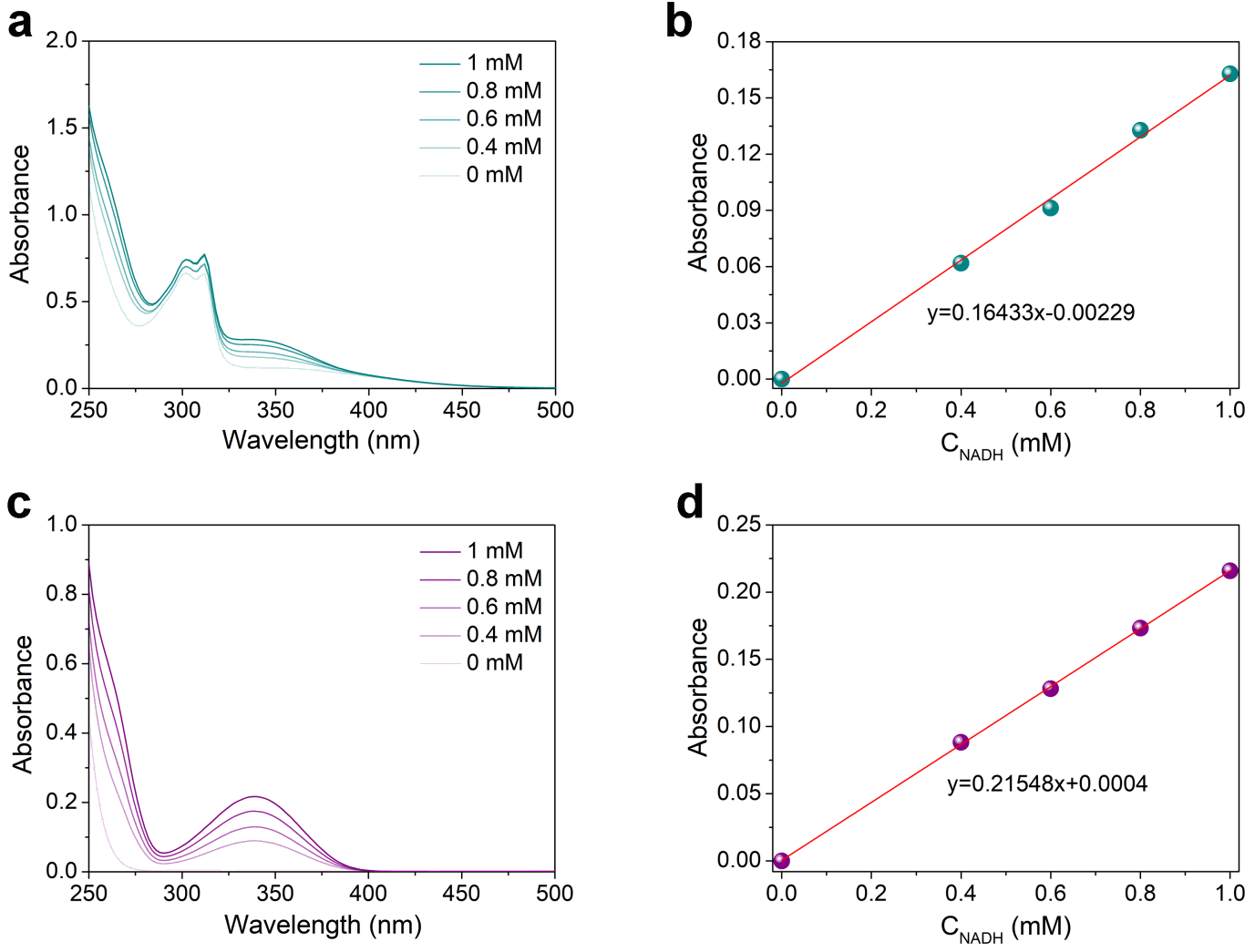


**Figure S18**: **NADH content calibration.** (a) UV-visible absorption spectra of of NADH at different concentrations in an environment containing an electron mediator and (b) the corresponding standard curve. (c) UV-visible light absorption spectra of NADH at different concentrations in an environment without electron mediator and (d) the corresponding standard curve.

**Table S1.** A comparative assessment of photocatalytic NADH regeneration efficiency was conducted between a microfluidic-based reaction system and conventional batch reactors.

| **Catalyst** | **Reaction time** | **Electron donor** | **[Cp*Rh(bpy)H_2_O]^2+^** | **NAD^+^ conversion rate (%)** | **Ref.** |
| --- | --- | --- | --- | --- | --- |
| BiOBr-180 | 126 s | TEOA | / | 100% | This work |
| RF@PANI-2 | 3 h | TEOA | Yes | 68.02 | 1 |
| COF-T3 | 20 min | TEOA | Yes | 89 | 2 |
| B-COF-2 | 10 min | TEOA | Yes | 5.5 | 3 |
| T-COF-2 | 10 min | TEOA | Yes | 74 | 3 |
| TMC* | 10 min | TEOA | Yes | 41 ± 0.7 | 4 |
| P25-TiO_2_ | 50 min | EDTA-4Na | Yes | 42 | 5 |
| ARTM36 | 50 min | EDTA-4Na | Yes | 76 | 5 |
| ARTM36 | 50 min | LA | Yes | 49 | 5 |
| ARTM36 | 50 min | TEOA | Yes | 39 | 5 |
| Ti_3_C_2_/COF-367 | 5 h | TEOA | / | 83.38 | 6 |
| Bi_2_MoO_6_ | 1 h | TEOA | / | 64.31 | 7 |
| Bi_2_MoO_6_ | 1 h | TEOA | Yes | 64.31 | 7 |
| Bi_12_O_17_Cl_2_–Bi_48_Al_2_O_75_–Al_2_O_3_ | 100 min | TEOA | / | 89 | 8 |
| Rh_m3_-N-PCN | 25 min | TEOA | / | ~66 | 9 |
| (MIL-125-Py-Rh)_0.05_ | 60 min | TEOA | / | 66.4 | 10 |
| TCM-15% | 30 min | TEOA | Yes | 95 | 11 |
| TCM-15% | 30 min | TEOA | / | 46 | 11 |
| g-C_3_N_4_@α-Fe_2_O_3_/C | 16 min | TEOA | Yes | 76.3 | 12 |

**References**

1. L. Zhou, Z. Su, J. Wang, Y. Cai, N. Ding, L. Wang, J. Zhang, Y. Liu, J. Lei, *Appl. Catal., B* **2024**, *341*, 123290.
2. L. Tong, Z. Gong, Y. Wang, J. Luo, S. Huang, R. Gao, G. Chen, G. Ouyang, *J. Am. Chem. Soc.* **2024**, *146*, 21025–21033.
3. Y. Wang, H. Liu, Q. Pan, C. Wu, W. Hao, J. Xu, R. Chen, J. Liu, Z. Li, Y. Zhao, *J. Am. Chem. Soc.* **2020**, *142*, 5958–5963.
4. Y. Sun, J. Shi, Z. Wang, H. Wang, S. Zhang, Y. Wu, H. Wang, S. Li, Z. Jiang, *J. Am. Chem. Soc.* **2022**, *144*, 4168–4177.
5. P. Wei, Y. Zhang, J. Dong, Y. Cao, S. M. Y. Lee, W. Lou, C. Peng, *Appl. Catal., B* **2024**, *357*, 124257.
6. J. Qu, T. Yang, P. Zhang, F. Yang, Y. Cai, X. Yang, C. M. Li, J. Hu, *Appl. Catal., B* **2024**, *348*, 123827.
7. Y. Chai, Z. Pang, H. Jiang, C. C. Tsoi, L. Wan, Y. Du, H. Jia, Y. Zhu, D. Liu, F. Xie, G. Zhou, X. Zhang, *Green Chem.* **2025**, *27*, 623–632.
8. Z. W. Wang, X. Tan, Z. H. Wu, J. H. Chen, Y. Z. Zhu, H. F. Wang, *J. Mater. Chem. A* **2025**, *13*, 8726–8733.
9. Y. Zhang, J. Liu, *Chem. Eur. J.* **2022**, *28*, e202201430.
10. G. Lin, Y. Zhang, Y. Hua, C. Zhang, C. Jia, D. Ju, C. Yu, P. Li, J. Liu, *Angew. Chem. Int. Ed.* **2022**, *61*, e202206283.
11. P. Wei, J. Dong, X. Gao, L. Chang, Z. Huang, H. Zheng, S. M. Y. Lee, W. Y. Lou, C. Peng, *ACS Sustainable Chem. Eng.* **2024**, *12*, 6881–6893.
12. Y. Wu, J. Ward-Bond, D. Li, S. Zhang, J. Shi, Z. Jiang, *ACS Catal.* **2018**, *8*, 5664–5674.
